# Metabolic potential of *Nitrososphaera*-associated clades

**DOI:** 10.1093/ismejo/wrae086

**Published:** 2024-05-14

**Authors:** Qicheng Bei, Thomas Reitz, Martin Schädler, Logan H Hodgskiss, Jingjing Peng, Beatrix Schnabel, François Buscot, Nico Eisenhauer, Christa Schleper, Anna Heintz-Buschart

**Affiliations:** Department of Soil Ecology, Helmholtz Centre for Environmental Research – UFZ, 06120 Halle (Saale), Germany; German Centre for Integrative Biodiversity Research (iDiv) Halle-Jena-Leipzig, 04103 Leipzig, Germany; Department of Biological Sciences, University of Southern California, Los Angeles, CA 90089, United States; Department of Soil Ecology, Helmholtz Centre for Environmental Research – UFZ, 06120 Halle (Saale), Germany; German Centre for Integrative Biodiversity Research (iDiv) Halle-Jena-Leipzig, 04103 Leipzig, Germany; German Centre for Integrative Biodiversity Research (iDiv) Halle-Jena-Leipzig, 04103 Leipzig, Germany; Department of Community Ecology, Helmholtz Centre for Environmental Research – UFZ, 06120 Halle (Saale), Germany; Archaea Biology and Ecogenomics Unit, Department of Functional and Evolutionary Ecology, University of Vienna, 1030 Vienna, Austria; College of Resources and Environmental Sciences, China Agricultural University, Beijing 100193, China; Department of Soil Ecology, Helmholtz Centre for Environmental Research – UFZ, 06120 Halle (Saale), Germany; Department of Soil Ecology, Helmholtz Centre for Environmental Research – UFZ, 06120 Halle (Saale), Germany; German Centre for Integrative Biodiversity Research (iDiv) Halle-Jena-Leipzig, 04103 Leipzig, Germany; German Centre for Integrative Biodiversity Research (iDiv) Halle-Jena-Leipzig, 04103 Leipzig, Germany; Institute of Biology, Leipzig University, 04103 Leipzig, Germany; Archaea Biology and Ecogenomics Unit, Department of Functional and Evolutionary Ecology, University of Vienna, 1030 Vienna, Austria; Swammerdam Institute for Life Sciences, University of Amsterdam, 1098 XH Amsterdam, the Netherlands

**Keywords:** ammonia-oxidizing archaea, Nitrososphaerales, 54d9, metagenomics

## Abstract

Soil ammonia-oxidizing archaea (AOA) play a crucial role in converting ammonia to nitrite, thereby mobilizing reactive nitrogen species into their soluble form, with a significant impact on nitrogen losses from terrestrial soils. Yet, our knowledge regarding their diversity and functions remains limited. In this study, we reconstructed 97 high-quality AOA metagenome-assembled genomes (MAGs) from 180 soil samples collected in Central Germany during 2014–2019 summers. These MAGs were affiliated with the order *Nitrososphaerales* and clustered into four family-level clades (NS-α/γ/δ/ε). Among these MAGs, 75 belonged to the most abundant but least understood δ-clade. Within the δ-clade, the *amoA* genes in three MAGs from neutral soils showed a 99.5% similarity to the fosmid clone 54d9, which has served as representative of the δ-clade for the past two decades since even today no cultivated representatives are available. Seventy-two MAGs constituted a distinct δ sub-clade, and their abundance and expression activity were more than twice that of other MAGs in slightly acidic soils. Unlike the less abundant clades (α, γ, and ε), the δ-MAGs possessed multiple highly expressed intracellular and extracellular carbohydrate-active enzymes responsible for carbohydrate binding (CBM32) and degradation (GH5), along with highly expressed genes involved in ammonia oxidation. Together, these results suggest metabolic versatility of uncultured soil AOA and a potential mixotrophic or chemolithoheterotrophic lifestyle among 54d9-like AOA.

## Introduction

Over the past two decades, research on ammonia-oxidizing archaea (AOA) has greatly enhanced our knowledge of the global nitrogen cycle [1–3]. Ammonia oxidation, the initial and rate-limiting step in nitrification, is performed by both archaea and bacteria. Although this process plays a crucial role in pristine ecosystems for cycling reactive nitrogen compounds, it poses significant environmental challenges in fertilized agricultural soils, where plants absorb <50% of inorganic fertilizers [4]. The excess becomes a substrate for microbial ammonia oxidation, resulting in the production of nitrate and the by-product N_2_O. Nitrate contributes to water eutrophication, whereas N_2_O acts as a potent greenhouse gas, both causing severe environmental issues [5]. To address and mitigate these challenges, itis crucial to gain a deeper understanding of the prevalent nitrifying microorganisms in soils.

Through the sequencing of *amoA* genes (encoding ammonia monooxygenase subunit A), researchers have identified AOA as widely distributed in both marine and terrestrial environments [6, 7]. Previous investigations of global *amoA* genes have revealed that 82% of soil AOA belong to the order *Nitrososphaerales* (NS), falling into six family-level clades designated by Greek letters (α, β, γ, δ, ε, and ζ) [8]. To date, only a few cultivated strains with completed genomes from the NS-α and NS-ζ clades have been obtained, including *Nitrososphaera viennensis* EN76 [9], *Candidatus* (*Ca*.) Nitrososphaera gargensis Ga9.2 [10], and *Ca*. Nitrososphaera evergladensis SR1 [11], as well as *Ca*. Nitrosocosmicus hydrocola G61 [12], *Ca*. N. franklandianus C13 [13], *Ca*. N oleophilus MY3 [14], and *Ca*. *N. arcticus* Kfb [15]. The analysis of these intact genomes has provided precise insights into core genomic features of soil NS, including ammonia oxidation and the 3-hydroxypropionate/4-hydroxybutyrate (3-HP/4-HB) cycle for fixing CO_2_ [16]. However, for the other NS clades, only metagenome-assembled genomes (MAGs) have been obtained.

Despite constituting more than one-third of soil AOA according to environmental surveys [8], the NS-δ clade is often represented by the 54d9 clone (submitted in 2004), obtained from a sandy ecosystem near Darmstadt, Germany [2]. 54d9-like AOA have been detected in various habitats worldwide, including forests, grasslands, agricultural soils, and the Arctic, through *amoA* gene sequencing [7, 17, 18]. These AOA are also frequently found in aquatic environments, such as rice paddies [19, 20], lakes [21], and river sediments [22, 23]. In addition, previous studies using ^13^CO_2_-DNA stable isotope probing (SIP) have indicated that the autotrophic growth of AOA in soils is primarily associated with NS-α rather than NS-δ [20, 24]. In a recent study, several NS-δ MAGs were assembled from UK river sediments, revealing the presence of carbohydrate-active enzymes (CAZymes) like GH5, GH130, and GH133, which are involved in carbohydrate degradation [25]. This suggests that NS-δ AOA may have the potential to adopt a mixotrophic (utilizing both inorganic and organic carbon sources)/heterotrophic lifestyle, potentially explaining their high abundance in global soils. However, detailed analyses of NS-δ AOA genome inventories are still scarce.

In this study, we present 97 high-quality NS MAGs obtained from 180 soil samples collected at the Global Change Experimental Facility (GCEF), an experimental field platform operated by the Helmholtz Centre for Environmental Research (UFZ) in Bad Lauchstädt, Germany, during the summers of 2014–2019 [26]. These MAGs were categorized into four clades: NS-α, NS-γ, NS-δ, and NS-ε. For comparative genomic analysis, the primary focus was on genes related to carbon and nitrogen metabolism across different NS clades. We conducted a comprehensive investigation of their genomic traits, gene expressions, and habitat preferences, and compared them to public MAGs (Supplementary Table S1). In addition, BLASTN searches of the *amoA* genes from our MAGs against NCBI databases were performed to assess their global distribution in previously published studies.

## Materials and methods

### Study site, soil sampling, and metagenomic sequencing

**Figure 1:**
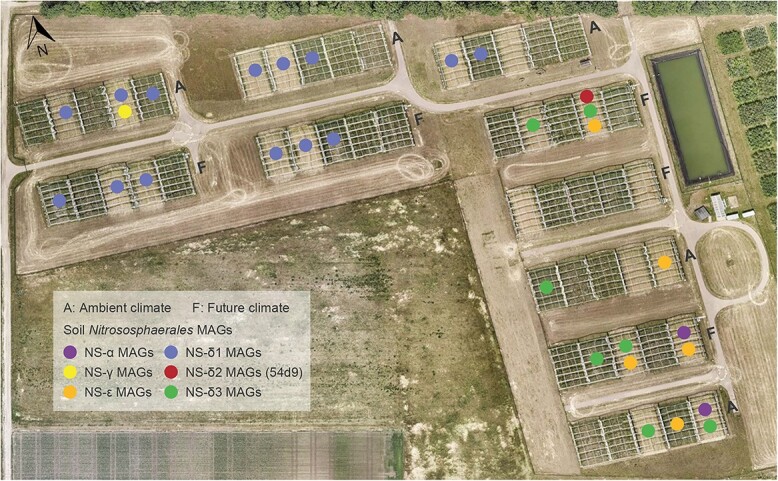
Layout of the GCEF research station and the plots from which the high-quality NS MAGs were retrieved; for land-use types and soil pH values, see Supplementary Fig. S1 and Supplementary Table S2; photo: UFZ.

The GCEF research platform of UFZ is located in Bad Lauchstädt, Saxony-Anhalt, Germany (51°23′30″N, 11°52′49″E, 116 m a.s.l.) [27]. The study area has a subcontinental climate with an average annual rainfall of 489 mm and a mean temperature of 8.9°C for the period 1896–2013. The GCEF was established to study the impacts of future climate on agricultural ecosystems across different land-use types (Fig. 1, Supplementary Fig. S1). The future climate scenario is characterized by a 20% reduction in rainfall during summer and a 10% increase in spring and autumn, coupled with ~0.55°C rise in mean daily temperature. To simulate future climate conditions, the roof and side panels automatically close from sunset to sunrise. The GCEF comprises 10 main plots, each containing five subplots randomly assigned to different land-use types. The soil at the GCEF is classified as Haplic Chernozem, characterized by a high content of organic carbon and water-holding capacity [28]. It is worth mentioning that the soils at the GCEF site exhibit a pH gradient ranging from 6.0 to 7.5 before the start of experiment (Supplementary Fig. S1).

As part of the previous study [26], we collected 180 soil samples (0–15 cm depth) from three land-use types: conventional farming, organic farming, and intensively managed grassland, during the 2014–2019 summers (2 climates × 3 land-use types × 5 replicates × 6 summers). Soil DNA extraction was carried out using the DNeasy PowerSoil kit (Qiagen) following the manufacturer’s instructions. The metagenomic library preparation and sequencing (2 × 150 bp) were performed by the NGS Competence Center Tübingen. More details regarding the GCEF, land-use regimes, soil properties and sequencing results (see previous Supplementary Table S4) have been described previously [26].

### Optimization of NS MAGs and functional annotation

In our previous study [26], metagenome assembly was conducted using MEGAHIT [29], and binning was performed using MetaBAT2 [30] within the metaWRAP v1.3 [31]. A total of 223 AOA MAGs were recovered from 180 soil samples (completeness >50% and contamination <10%). To enhance the quality of MAGs, particularly those belonging to the NS-δ2 and NS-γ clades (completeness <85%), the samples were individually assembled with metaSPAdes [32] within the metaWRAP (−m 900, −t 30, ∓metaspades). Metagenome binning was reconstructed individually using MetaBAT2 [30], MaxBin2 [33], and SemiBin2 [34]. The resulting bins from the three tools were consolidated into a single bin set for each sample using the Bin_refinement function within metaWRAP. The quality of MAGs was evaluated using CheckM v1.2.0 [35], and the taxonomy of MAGs was assigned using GTDB-Tk v2.0.0 [36] with the R07-RS207 database. For each soil sample, only the highest quality of MAG from each NS clade was retained for downstream analysis. Genomes affiliated with class *Nitrososphaeria* were also downloaded from the National Center for Biotechnology Information (NCBI) database (until June 2023) (Supplementary Table S1). Gene predictions for MAGs were performed using Prodigal v2.6.3 [37]. Protein functions were annotated using the Kyoto Encyclopedia of Genes and Genomes server (BlastKOALA/GhostKOALA) [38]. CAZymes were annotated based on the dbCAN-HMMdb-v12 database using the dbCAN3 server (E-Value <1e-15, coverage >0.80) [39]. To predict the signal peptides and transmembrane helices of CAZymes, the SignalP v6.0 [40] and DeepTMHMM v1.0.24 [41] servers were applied, respectively. The protein structures of selected CAZymes were predicted using AlphaFold2 within ColabFold [42].

### Phylogenetic and global distribution analysis of AOA MAGs

Phylogenomic analysis of MAGs and reference genomes was performed based on the concatenated proteins from CheckM. The protein sequences were aligned using MUSCLE v3.8.1551 [43] and subsequently trimmed with trimAl v1.2 using the automated1 setting [44]. A phylogenetic tree was generated based on maximum likelihood estimation using RAxML v8.2.12 (-f a -m PROTGAMMAWAG -N 1000) [45]. The average nucleotide identity (ANI) among the genomes was calculated using Pyani v0.2.12 with the ANIb algorithm [46].

For the phylogenetic analysis of *amoA* genes, representative sequences from Alves *et al*. [8] were downloaded and aligned using MUSCLE. The phylogenetic tree based on the aligned *amoA* genes was generated using RAxML (-f a -m GTRGAMMA -N 1000). The 16S rRNA genes in MAGs were identified using the ssu_finder module in CheckM. All phylogenetic trees were visualized and edited using the iTOL webserver (https://itol.embl.de/). To evaluate the global distribution and occurrence of the *amoA* genes found in our MAGs, a BLASTN search (identity >96%, query coverage >90%) was conducted against the NCBI’s GenBank.

### Soil metatranscriptome and mapping of AOA MAGs

The preparation and sequencing of soil metatranscriptomic libraries have been described previously [26]. In May and July 2022, surface soils (0–10 cm depth) were collected from conventional farming at the GCEF (Supplementary Fig. S1). At each time point, 12 soil samples were taken from the ambient and future climate plots. The soil samples were flash-frozen in liquid nitrogen in the field, and stored at −80°C in the refrigerator until RNA extraction. Sequencing yielded on average 1.6 (sd = 1.0) and 2.0 (sd = 1.0) million paired-end mRNA reads per sample for the May and July sampling campaigns, respectively.

The relative abundances of MAGs across 180 metagenome and 24 metatranscriptome were determined using CoverM v0.4.0 in genome mode (bwa-men, --min-read-percent-identity 0.97 --min-read-aligned percent 0.90) (https://github.com/wwood/CoverM). Gene expression levels were determined by mapping of mRNA reads against the encoded genes in each MAG using CoverM in contig mode (bwa-men, --min-read-percent-identity 0.97 --min-read-aligned percent 0.90). The MAG abundances and gene expression levels were reported as reads mapped per kilobase per million mapped reads (RPKM) values. The expression level of selected genes was log_2_ (RPKM+1) transformed. Differences in abundance between treatments were tested by one-way ANOVA and *P* < .05 was considered statistically significant.

## Results and discussion

### Phylogeny and relative abundance of soil AOA MAGs

**Figure 2:**
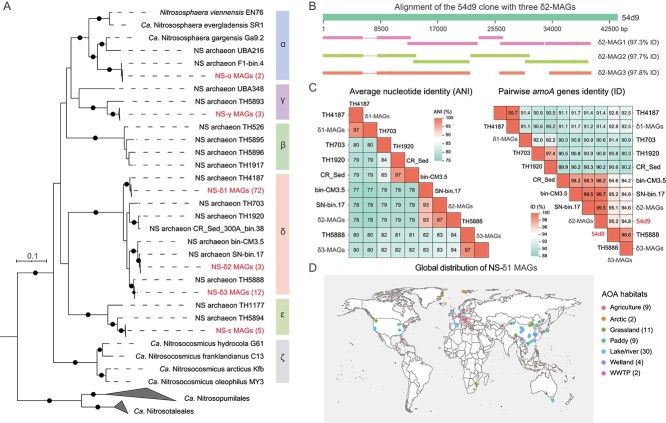
Phylogenomic and comparative analysis of recovered AOA MAGs; (A) maximum-likelihood phylogenomic tree of recovered NS MAGs and reference genomes; the tree was inferred from concatenated phylogenetic markers and rooted with the *Ca*. Nitrosocaldales strains 3F and SCU2; the black dots represent bootstrap values > 90%; (B) alignment of the 54d9 clone and three δ2-MAGs in this study; (C) pairwise ANI and *amoA* genes of recovered δ-MAGs and reference genomes; (D) global distribution of the NS-δ1 MAGs based on the BLASTN of *amoA* genes; for *amoA* gene sequences of our MAGs, see Supplementary Files; WWTP, wastewater treatment plant.

In contrast to our previous study [26], after the optimization process, the average completeness of δ1-, δ3-, and ε-MAGs increased from 96.8%, 93.9%, and 92.6% to 98.3%, 95.8% and 94.7%, respectively. The use of metaSPAdes combined with MetaBAT2 yielded the highest-quality MAGs for the NS-δ2 (completeness >95%) and NS-γ (completeness >85%) clades. Finally, we obtained 97 high-quality (completeness >85% and contamination <5%) (Fig. 1, Table 1 and Supplementary Table S2) and 85 medium-quality (completeness >80% and contamination <10%) MAGs (Supplementary Fig. S2, Supplementary Table S3). Based on the genome-wide taxonomic ranking [25], the high-quality AOA MAGs were clustered into four family-level clades: NS-α, NS-γ, NS-δ, and NS-ε (Fig. 2A). Although ammonia-oxidizing bacteria (e.g. *Nitrosomonas*, *Nitrosospira*) were detected across all soil samples (Supplementary Fig. S3), the corresponding MAGs were not recovered. Regarding genome size, the α-/δ-MAGs were much smaller compared to the three NS-α representatives (*N. viennensis*, *Ca*. N. gargensis, and *Ca*. N. evergladensis) (2.52–2.95 Mb) (Supplementary Table S4), suggesting the loss of certain functions [10]. The clustering observed in the phylogenomic tree (Fig. 2A) generally aligns with phylogenetic trees inferred from the *amoA* (Supplementary Figs S4 and S5A) and 16S rRNA genes (Supplementary Fig. S5B) identified in assembled AOA MAGs.

**Table 1:** General information on the 97 NS MAGs recovered in this study.

**Lineages**	**No. of MAGs**	**Completeness (%)**	**Contamination (%)**	**GC (%)**	**Size (Mbp)**	**Genes**
Alpha	2	93.62 ± 0.11	3.08 ± 1.61	44.35 ± 0.03	1.38 ± 0.04	1689 ± 18
Gamma	3	86.74 ± 1.35	4.18 ± 0.21	38.05 ± 0.11	2.49 ± 0.05	3157 ± 103
Delta1	72	98.28 ± 0.77	1.49 ± 0.73	35.92 ± 0.04	1.95 ± 0.06	2403 ± 78
Delta2	3	96.19 ± 1.03	2.54 ± 0.95	35.70 ± 0.04	1.80 ± 0.15	2277 ± 213
Delta3	12	95.78 ± 1.41	2.13 ± 0.93	35.35 ± 0.07	1.64 ± 0.08	2016 ± 119
Epsilon	5	94.70 ± 2.50	2.62 ± 0.88	28.99 ± 0.08	2.16 ± 0.09	2550 ± 105

Values are reported as the mean ± standard deviation. Detailed genomic statistics of the MAGs are presented in Supplementary Table S2.

The majority of our MAGs (87 out of 97) were categorized into three delta sub-clades: NS-δ1, NS-δ2, and NS-δ3. The δ1-MAGs were obtained from the upper left field, whereas δ2- and δ3-MAGs were recovered from the right field at the GCEF (Fig. 1). The δ1-MAGs were consistently assembled every summer from 2014 to 2019, resulting in a total of 72 MAGs. These MAGs were clustered with the TH4187 MAG [25] and showed ANI values below 80% (Supplementary Fig. S6, Supplementary Table S5) and *amoA* gene similarities below 93% when compared to other δ-MAGs (Supplementary Fig. S7). We observed a significant sequence similarity of 97.6% between the 54d9 clone (based on full-length) and three δ2-MAGs (Fig. 2B). In addition, when examining the marker gene *amoA*, a similarity of 99.5% between the 54d9 clone and the δ2-MAGs was observed. This suggests that the δ2-MAGs can serve as representative genomic models for the 54d9 clone, offering initial insights into its metabolic potential. Although the *amoA* genes of δ2-MAGs were detected in several plots (Supplementary Fig. S8), three δ2-MAGs were consistently recovered from a single plot over three summers. The δ3-MAGs exhibited clear genetic differentiation from the δ2-MAGs, as demonstrated by an ANI of 84% and an *amoA* gene identity of 94.6% (Fig. 2C, Supplementary Figs S6 and S7). Given that species delineation is often defined based on ANI values of ≥95% [47], the δ3-MAGs may belong to a distinct genus. The ANI values among TH703, TH1920, and TH5888 MAGs [25] were also below 95%, indicating a high genetic diversity at the species level within the NS-δ clade (Fig. 2C, Supplementary Fig. S6). However, the recovered MAGs do not represent the overall diversity of NS AOA in the GCEF soils, as genomes containing *amoA* genes with low coverage remained unassembled (Supplementary Fig. S8).

**Figure 3:**
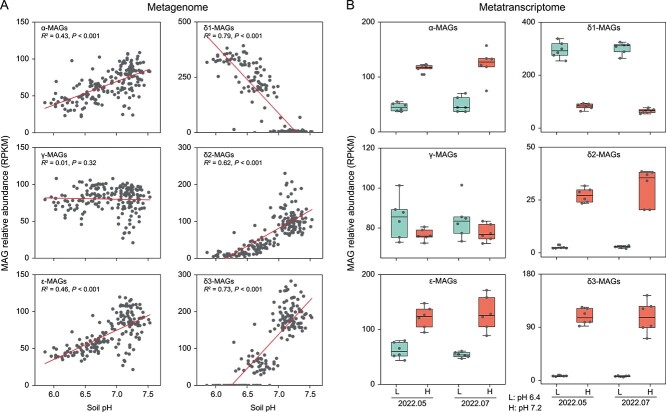
Metagenomic and metatranscriptomic abundance of recovered AOA MAGs; (A) metagenomic abundance of recovered MAGs across 180 soil samples during the 2014–2019 summers; lines indicate the Pearson correlation between soil pH and MAG relative abundance in metagenomic datasets; (B) metatranscriptomic abundance of recovered MAGs across 24 soil samples in May and July 2022; boxplots show median, upper and lower quartile, and minimum and maximum values; L and H denote groups of plots with mean soil pH values of 6.4 and 7.2, respectively.

Metagenomic mapping revealed a significant correlation between the relative abundance of α-/δ-/ε-MAGs and soil pH during the summers of 2014–2019 (Fig. 3A). The δ1-MAGs exhibited a significantly higher abundance in slightly acidic soils compared to other MAGs. This observation is consistent with the metatranscriptomic mapping, which showed that the activities of δ1-MAGs were higher in soils with a mean pH of 6.4 compared to those with a mean pH of 7.2 (Fig. 3B). Soil pH is a critical factor in shaping the distribution and activity of AOA [48], with only a few clades capable of thriving in acidic soils, such as the *Nitrosotalea* lineage [49]. The genomic and transcriptomic findings suggest that δ1-MAGs prevailed in the slightly acidic soils.

### BLASTN analysis of MAG *amoA* genes in previous studies

The BLASTN analysis identified a total of 1681, 2747, and 1501 hits with high similarity (> 96%) to the *amoA* genes derived from δ1-, δ2-, and δ3-MAGs, respectively (Supplementary Tables S6–S8). We found that the *amoA* genes of δ1-MAGs were detected in 77 published studies across 16 countries (Fig. 2D), primarily in agricultural soils and lake/river sediments. Hotspot areas such as Eastern Washington, USA [50], the Inner Mongolia Grassland [51], the Qinghai-Tibet Plateau in China [52], agricultural soils in lower Austria [53], and Arctic soils [18] showed the presence of all three sub-clades of δ-MAGs. Compared to the δ-MAGs, the α- (53), γ- (150), and ε-MAGs (310) (Supplementary Tables S9–S11) showed fewer BLASTN hits, likely due to the choice of primers for amplification. A bias was observed in the commonly used *amoA* primers amoAF/amoAR [54], as they exhibit 3, 2, 4, and 5 bp mismatches with the *amoA* genes from α-, β- [25], γ-, and ε-MAGs, respectively (Supplementary Fig. S9). As more AOA MAGs are assembled, our results suggest the need for developing new primers targeting different NS clades.

The δ1-MAGs were obtained from soil samples with a wide range of NH_4_^+^ concentrations at the GCEF, ranging from 1 to 22 mg kg^−1^ [26]. Previous research has shown that the addition of KNO_3_, NH_4_NO_3_, and [NH_4_]_2_SO_4_ did not significantly impact the copy numbers of δ1-like *amoA* genes (> 98% similarity) in agricultural soils (pH 5.67) in Purkersdorf, Austria [53]. Similarly, δ1-like *amoA* genes were detected in both control and fertilized soils (pH 5.24) in Paterson, Washington, USA [50]. In addition, the δ1-like *amoA* genes were also frequently detected in nutrient-enriched wastewaters [55, 56]. Based on the BLASTN hits (Supplementary Table S6), it appears that δ1-MAGs can adapt to varying levels of NH_4_^+^ content. Although the cultured representative (*N. viennensis*) is an autotrophic ammonia oxidizer, δ1-like *amoA* genes were exclusively observed in the light DNA and RNA fractions of ^13^CO_2_-SIP after 8 and 12 weeks of incubation [57] (Supplementary Fig. S10, Supplementary Table S12). In contrast, the *amoA* genes present in the heavy RNA-SIP fractions showed a 99.8% similarity to the NS-β TH526 MAG after 8 weeks of incubation. These findings, along with previous studies [18, 58, 59], suggest a potential mixotrophic/heterotrophic lifestyle of soil NS-δ AOA.

The relative abundance of *amoA* genes affiliated with the 54d9 clone was observed to significantly decrease in grassland soils with nitrogen fertilizer, whereas *Nitrosocosmicus* AOA (fosmid clone 29i4) [14] were enriched [60]. In this study, three δ2-MAGs were recovered from a single plot with low ammonia content (average 1.2 mg kg^−1^) (Fig. 3B). The *amoA* genes derived from γ-MAGs were previously detected in river sediments and grassland soils (Supplementary Table S10) [8]. Our γ-MAGs were assembled from slightly acidic soils, which is similar to their detection in Paterson soils (pH 5.24) in Washington, USA [50]. The *amoA* genes derived from ε-MAGs were mainly detected in agricultural soils, grasslands, and sediments (Supplementary Table S11). Thus, the distribution of different clades of NS AOA may be related to soil properties and their lifestyles.

### Nitrogen metabolism and gene expression of soil AOA MAGs

**Figure 4:**
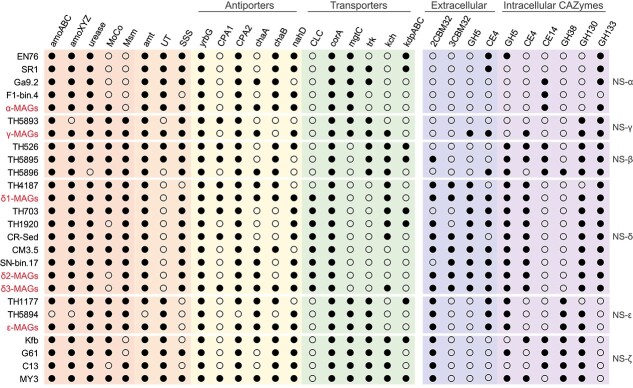
Metabolic potential of AOA genomes; the presence and absence of selected genes are indicated by a filled or empty circle, respectively; Amo, ammonia monooxygenase; MoCo, molybdenum cofactor; Msm, multiple sugar metabolism (ATP-binding protein); Amt, ammonium transporter; UT/SSS, urea transporters; YrbG, Ca^2+^/Na^+^ antiporter; CPA, cation/proton antiporter; Cha, Na^+^(Ca^2+^)/H^+^ antiporter; NahD, Na^+^/H^+^ antiporter; CLC, Cl^-^ channels; CorA/MgtC, mg^2+^ transport system; Trk/Kch, K^+^ transport system; KdpABC, K^+^ transport system; CBM, carbohydrate-binding module; GH, glycoside hydrolase; CE, carbohydrate esterase.

**Figure 5:**
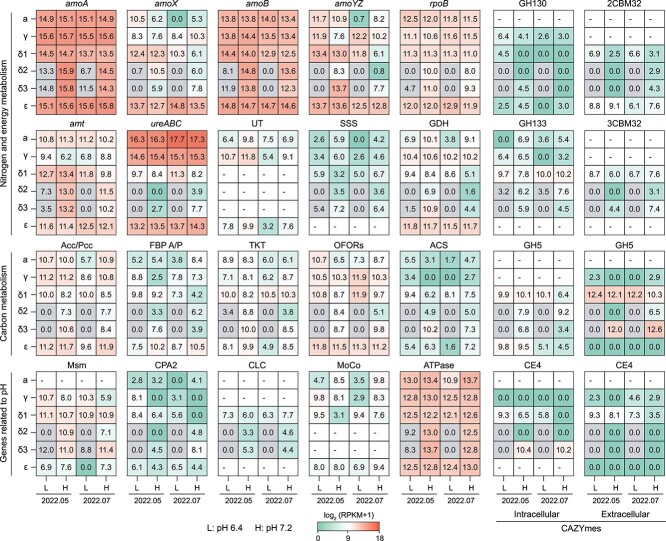
Expression levels of selected genes in recovered AOA MAGs; mean values were reported only if they were observed in at least two out of six replicates; dashes represent the absence of the selected gene in MAGs; a grey background indicates genes in MAGs that may lack sufficient coverage from metatranscriptome sequencing; L and H denote groups of plots with mean soil pH values of 6.4 and 7.2, respectively; GDH, glutamate dehydrogenase; Acc/Pcc, acetyl-CoA/propionyl-CoA carboxylase; FBP A/P, fructose 1,6-bisphosphate aldolase/phosphatase; TKT, transketolase; OFORs, 2-oxoacid: ferredoxin oxidoreductases; ACS, acetyl-CoA synthetase.

Despite the significant variation in genome size, the NS MAGs appear to be functionally conserved in their ability to utilize ammonia and urea as nitrogen and energy sources (Fig. 4 and Supplementary Fig. S11, Supplementary Tables S13 and S14). Ammonia oxidation is a core genomic feature of AOA and plays a vital role in energy metabolism [9], as evidenced by the high expression levels of *amoABC* and *amt* transporter genes (Fig. 5). The recently confirmed *amoX* and two newly proposed subunits, *amoY* and *amoZ* [61], were also detected in our MAGs. Consistent with previous observations [61], the *amoC* gene displayed the highest expression among all subunits (Supplementary Fig. S12). The copper-containing nitrite reductase gene (*nirK*) was also highly expressed, although its precise function remains unclear (Supplementary Fig. S12). The low expression levels of *amoXYZ* and RNA polymerase subunit B (*rpoB*) genes in δ2- and δ3-MAGs under relatively low soil pH may suggest a lack of metatranscriptome sequencing coverage (Fig. 5).

Some AOA are also capable of using urea as an energy source, possessing genes encoding a urease gene cluster (*ureABCDEFG*) and two adjacent urea transporters (UTs and sodium-solute symporter [SSS]) [16]. Functional annotation revealed that, although all δ-MAGs contain genes encoding urease, only one low affinity SSS gene was detected. A recent study also found that UT was absent in the NS-δ MAGs assembled from soils in South Florida, USA [62]. The SSS transporter was closely clustered with agmatinase (SpeB) in the NS-δ clade (Supplementary Fig. S11), catalyzing the conversion of agmatine to putrescine and urea. This enzyme was conserved in all MAGs and also expressed, suggesting it may play an important role in the regulation of urea concentration [63]. In addition, the ammonia and sulfur assimilation machinery, such as glutamate dehydrogenase, glutamine synthetase, and sulfite reductase, was conserved among all the MAGs and highly expressed (Fig. 5, Supplementary Figs S12 and S13).

The expression level of *ureABC* genes in δ-MAGs was significantly lower compared to other MAGs, whereas the *amt* gene showed relatively high expression levels, indicating that NS-δ AOA primarily rely on the presence of ammonia as their main energy source. The hydrolysis of urea has been reported as a mechanism for obligate acidophilic *Nitrosotalea devanaterra*-like AOA in acidic tea orchard soil (pH 3.75) and forest soil (pH 5.4) [64], which could also be a strategy utilized by NS-δ AOA. In the δ-MAGs, only the *ureABC* derived from δ1-MAGs was found to be highly expressed in slightly acidic soils, suggesting that urea hydrolysis may provide additional ammonia to support their growth.

### Carbon metabolism and gene expression of soil AOA MAGs

The 3-HP/4-HB cycle for CO_2_-fixation was encoded in all AOA MAGs [65], and the key enzyme acetyl-CoA/propionyl-CoA carboxylase (Acc/Pcc) exhibited active expression (ε-MAGs > γ-MAGs > α-MAGs > δ-MAGs) (Fig. 5, Supplementary Fig. S14). Although a carbonic anhydrase homolog was present in the three NS-α representatives, it was absent in our MAGs [16]. Past studies have often linked the nitrification activity of soil AOA to the NS-α clade using the ^13^CO_2_-SIP technique [20, 24]. The relatively low expression levels of ACC/PCC in δ-MAGs, especially in slightly acidic soils, may explain their absence in heavy ^13^CO_2_-SIP fractions [57]. However, the function of *acc*/*pcc* genes from δ-MAGs in soils with a mean pH of 7.2 remains to be determined [66] (Fig. 5). The expression results also support a recent study [62], suggesting that the NS-ε clade may significantly contribute to soil ammonia oxidation.

Consistent with a previous study [25], several key genes involved in glycolysis were absent in all AOA MAGs and representative genomes, including glucose-6-phosphate isomerase, phosphofructokinase, and pyruvate kinase (PK) (Supplementary Fig. S15, Supplementary Tables S13 and S14). Only *Ca*. N. gargensis and α-MAGs were found to have a pyruvate phosphate dikinase for the conversion between phosphoenolpyruvate (PEP) and pyruvate [10]. Nonetheless, all MAGs encoded and expressed fructose 1,6-bisphosphate aldolase/phosphatase (PBP A/P) (Fig. 5). As previously reported, PBP A/P is regarded as an ancestral gluconeogenic enzyme in archaea [67], which aligns well with the expression patterns of transketolase in the non-oxidative pentose phosphate pathway (Fig. 5). This supports carbon flow from the TCA cycle to gluconeogenesis, facilitated by phosphoenolpyruvate carboxykinase converting oxaloacetate to PEP (Supplementary Fig. S15). Furthermore, most MAGs, except for α-MAGs, were equipped with the multiple sugar metabolism (Msm) system (Supplementary Fig. S11). This system is associated with the uptake of various sugars [68], and the high expression activities likely enhance the assimilation of carbon compounds.

Acetyl-coenzyme A (acetyl-CoA) is a central metabolic intermediate in carbon metabolism of AOA MAGs, participating in gluconeogenesis, 3-HP/4-HB, and the TCA cycle (Supplementary Figs S14–S16). The alpha and beta subunits of 2-oxoacid: ferredoxin oxidoreductases are present and actively expressed in all MAGs [16], enabling the conversion of acetyl-CoA/succinyl-CoA and CO_2_ to pyruvate, and using ferredoxin as a reducing agent [69]. In addition, all MAGs contain the acetyl-CoA synthetase (ACS) gene (Fig. 5), with the highest expression level observed in the δ-MAGs, suggesting the potential utilization of acetate. Unlike marine AOA [70], soil AOA MAGs lack the phosphotransacetylase-acetate kinase (AckA-Pta) pathway and lactate racemase (Lar), indicating their inability to perform fermentation.

### CAZymes and expression of soil AOA MAGs

The number of CAZymes in the *Nitrososphaeraceae* family exceeds those in the *Nitrosopumilaceae* and *Nitrosocaldaceae* (GTDB classification) [25], particularly in the NS-δ MAGs (~7 GHs per genome) (Supplementary Tables S15 and S16). All our δ-MAGs contain intracellular CE4, GH130, and GH133 enzymes, and these were actively expressed (Fig. 5). CE4 esterases are known for their involvement in chitin and peptidoglycan degradation [71], leading to the release of acetate products [72]. As previously mentioned, the high activity of ACS in the δ-MAGs could facilitate the synthesis of acetyl-CoA from released acetate, thereby providing them with additional carbon sources. The GH130 family contains inverting phosphorylases that act on β-mannosides, whereas the GH133 family includes debranching amylo-α-1,6-glucosidase. In addition, these MAGs encoded multiple GT2 and GT4 genes, potentially involved in polysaccharides and biofilm formation [16].

**Figure 6:**
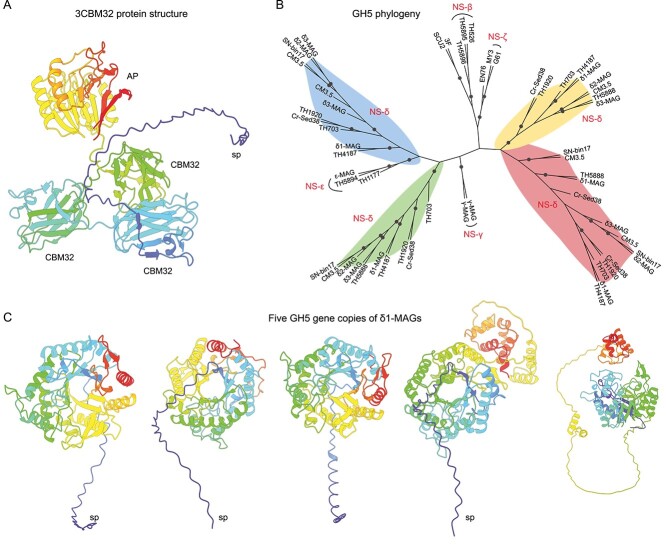
Phylogeny and predicted protein structure of CAZymes in recovered AOA MAGs; (A) protein structure of the 3CBM32 gene in NS-δ1 MAGs as predicted by AlphaFold2; (B) a maximum-likelihood tree of GH5 genes encoded by AOA genomes/MAGs; four GH5 gene clusters derived from δ-MAGs were colored; bootstrap values higher than 90% are indicated; (C) AlphaFold protein structures of the five GH5 gene copies in NS-δ1 MAGs; AP, acid phosphatase; SP, signal peptide; for GH5 and 3CBM32 gene sequences of the NS-δ1 MAGs, see Supplementary Files.

Only δ-MAGs displayed multiple highly expressed extracellular (with signal peptides) CE4, CBM32, and GH5 genes (Fig. 5). CBM32 is a carbohydrate-binding protein, also annotated as F5/8 type C domain, which is involved in binding a wide variety of carbohydrates (Supplementary Fig. S17). Among the δ-MAGs, 86%, 33%, and 100% of δ1-, δ2-, and δ3-MAGs contained three contiguously arranged CBM32 modules, respectively (Fig. 6A). When comparing the amino acid (AA) sequences, the three tandem CBM32s showed high identity within each sub-clade (δ1: 44%–59%; δ2: 49%–64%; δ3: 41%–63%). In addition, around 32% of δ1-MAGs and all ε-MAGs had two contiguously arranged CBM32 modules. The expression levels of 2CBM32 and 3CBM32 modules in δ1-MAGs were relatively high, especially in slightly acidic soils (Fig. 5). The protein structure of 3CBM32, as predicted by AlphaFold2, revealed a typical β-sandwich fold (Fig. 6A), with three residues may facilitate targeting substrates by creating a sugar-binding pocket [73]. At the C-terminal tail of 3CBM32, an acid phosphatase (AP) was identified (Fig. 6A), which catalyzes the hydrolysis of phosphate esters under acidic conditions [74]. Given that slightly acidic soils contain lower available phosphorus compared to neutral soils [26], the high activity of AP may enhance phosphate availability to δ1-MAGs.

Cellulases from glycoside hydrolase family 5 (GH5) are essential endoglucanase enzymes involved in breaking down various polysaccharides. Multiple copies of intracellular (1 ~ 2) and extracellular (3 ~ 4) GH5 genes were detected in our and public δ-MAGs (Supplementary Tables S15 and S16). These putative GH5s showed a high degree of identity within each sub-clade (δ1: 46%–65%; δ2: 49%–56%; δ3: 47%–88% AA). A phylogenetic analysis revealed four well-supported branches of GH5s within the δ-MAGs (Fig. 6B). Only one GH5 was encoded by γ- and ε-MAGs. AlphaFold2 structure prediction indicated that all GH5 enzymes possess a typical (β/α)_8_-barrel fold at the N-terminal domain [75] (Fig. 6C). Within the CAZymes, GH5 and CBM32 were highly expressed by δ1-MAGs (Fig. 5). GH5 and CBM32 are known to be co-expressed and work synergistically during the hydrolysis of mannans by *Clostridium thermocellum* [76, 77]. Considering the absence of surface dockerin in the GH5 proteins, it is reasonable to deduce that δ-MAGs may simultaneously secrete GH5 and CBM32 when the surrounding environment has a high carbohydrate concentration.

### Expression of pH and stress-related genes of soil AOA MAGs

Comparative analysis revealed several genomic traits that may distinguish the expression levels of recovered AOA MAGs (Fig. 4, Supplementary Fig. S18). Na^+^/H^+^ antiporters are important membrane transport proteins for maintaining the internal pH, cell volume, and Na^+^ concentration of living cells [78]. The cation/proton antiporter (CPA) superfamily mediates the exchange of monovalent cations, mainly Na^+^ and K^+^ [79]. It has been observed that CPA1 transporters in *Escherichia coli* were downregulated to prevent the cytoplasm from becoming too alkaline, whereas CPA2 transporters were downregulated to prevent overacidification [80]. The CPA1 genes from δ1/δ3-MAGs showed homology to the genes found in *Ca*. Nitrosocosmicus oleophilus (51% AA) [14] and *Ca*. Nitrosopolaris wilkensis (60% AA) [81], which are known to grow in soils with low pH. Two putative voltage-gated chloride channel (CLC) proteins were exclusively found in δ-MAGs, including the 54d9 clone (81% AA) (Fig. 4). CLC proteins have also been detected in thermoacidophilic and deeper-branching non-ammonia oxidizers, such as *Conexivisphaerales* [82] and *Geothermarchaeales* [83] (Supplementary Table S13). Previous studies have shown that *E. coli* utilized CLC channels to passively transport Cl^−^ ions under extreme acid conditions (pH 2.5) [84]. Other noteworthy proteins include Na^+^ antiporters (ChaAB, YrbG, and NhaD), Mg^2+^ (CorA, MgtABC), and K^+^ (Trk and Kdp) transporters (Supplementary Fig. S12), which may also enhance osmotic stress tolerance [24].

The expression levels of stress-related proteins, such as heat shock proteins (Hsp20, CCT), chaperones (DnaJ, DnaK, GrpE), superoxide dismutase (Fe/Mn-SOD), thioredoxin (TrxA), and compatible solute mannosylglycerate (MPGS), were ~10-fold higher than those of antiporters and transporters (Fig. 5, Supplementary Fig. S18). Consistent with previous observations [25], a cluster of genes involved in molybdenum cofactor (MoCo) biosynthesis were detected in NS MAGs but not in the three representatives (Fig. 4 and Supplementary Fig. S11). The biosynthesis of MoCo is an ancient and highly conserved pathway [85], which may provide additional protection against oxidative stress [25]. The expression levels of above-mentioned transporters were considerably lower compared to ATPase operons (Fig. 5), suggesting that ATPase may play a vital role for the soil AOA under osmotic stress [49].

## Conclusions

**Figure 7:**
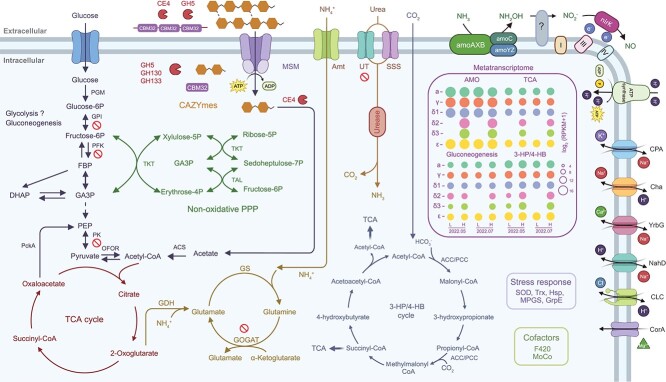
Metabolic reconstruction and gene expression of major pathways in NS-delta AOA MAGs; mean values were reported only if they were observed in at least two out of six replicates; PGM, phosphoglucomutase; GPI, glucose-6-phosphate isomerase; PFK, phosphofructokinase; PK, pyruvate kinase; PckA, phosphoenolpyruvate carboxykinase; TAL, transaldolase; GDH, glutamate dehydrogenase; GS, glutamine synthetase; GOGAT, glutamate synthase; NirK, nitrite reductase; SOD, superoxide dismutase; Trx, thioredoxin; Hsp, heat shock protein; MPGS, mannosyl-3-phosphoglycerate synthase; GrpE, molecular chaperone.

In conclusion, this study reveals a high degree of phylogenetic and metabolic diversity among soil AOA in the GCEF soils, particularly within the NS-δ clade. BLASTN analysis of *amoA* gene sequences strongly supports the global distribution of these 54d9-like AOA and confirms the dominant abundance of NS-δ MAGs among soil AOA lineages. The genome and expression studies presented here indicate differences in their metabolic potential compared to other lineages. Although NS-δ MAGs exhibit (like other AOA) high expression levels of genes involved in ammonia oxidation, carbon fixation, and central metabolism (AMO, TCA, 3-HP/4-HB, and gluconeogenesis), they lack the traditional UT, which might reduce their ability to utilize urea in soils (Fig. 7). We also identified multiple gene copies and observed high transcription levels of genes involved in utilization of organic carbon as energy source and for assimilation. Those encode enzymes involved in the degradation of carbohydrates (extracellular CBM32 and GH5), sugar import (via the Msm system), and high expression of genes involved in acetate metabolism. The consistent presence of these genes across all MAGs in the δ-clade, along with previous genomic [25] and SIP [57] studies, suggests that these organisms might rely on organic carbon either in a facultative heterotrophic or mixotrophic lifestyle or as chemolithoheterotrophs, generating energy via ammonia oxidation while assimilating carbon from organic sources. Collectively, our findings provide new insights into the metabolic potential of soil δ-AOA, offering genomic traits for their enrichment and isolation in future studies. Given the significant environmental impact of AOA through competition with plants for fertilizer and transformation of reactive nitrogen species into water-soluble nitrate, it will be important to identify effective and specific inhibitors targeting this least-understood but quantitatively important AOA clade in global soils.

## Supplementary Material

ISME_Supplemental_Figures_R3_wrae086

ISME_Supplemental_Tables_R3_wrae086

## Data Availability

The metagenomic and metatranscriptomic datasets have been deposited on the NCBI website under accession numbers PRJNA838942 and PRJNA903142, respectively. The 97 high-quality MAGs can be download from the NCBI website under accession numbers SAMN35131934 to SAMN35132030. The 85 medium-quality MAGs can be downloaded from the FigShare website (10.6084/m9.figshare.24421762).
