# Supplementary material for: Metabolic potential of Nitrososphaera-associated clades
Source: ISME J. 2024 May 14;18(1):wrae086. doi: 10.1093/ismejo/wrae086 (PMC11131427; doi:10.1093/ismejo/wrae086)

**Supplementary information for**

**Metabolic potential of *Nitrososphaera*-associated clades**

Qicheng Bei^1, 2, †, *^, Thomas Reitz^1, 2^, Martin Schädler^2, 3^, Logan H. Hodgskiss^4^, Jingjing Peng^5^, Beatrix Schnabel^1^, François Buscot^1, 2^, Nico Eisenhauer^2, 6^,

Christa Schleper^4^, Anna Heintz-Buschart^7, *^

***Corresponding authors:**

Qicheng Bei, Email: qicheng.bei@ufz.de

Anna Heintz-Buschart, Email: a.u.s.heintzbuschart@uva.nl

^†^Present address: Department of Biological Sciences, University of Southern California, Los Angeles, CA 90089, United States

**This file includes:**

Supplementary Fig. S1–18

Representative *amoA* gene sequences in recovered AOA MAGs

Representative GH5 and CBM32 genes in recovered AOA MAGs

Appendix 1: Function of selected KEGG Orthologs (KOs)

Appendix 2: Function of selected glycoside hydrolases (GHs)

Appendix 3: An overview of the bioinformatic pipeline


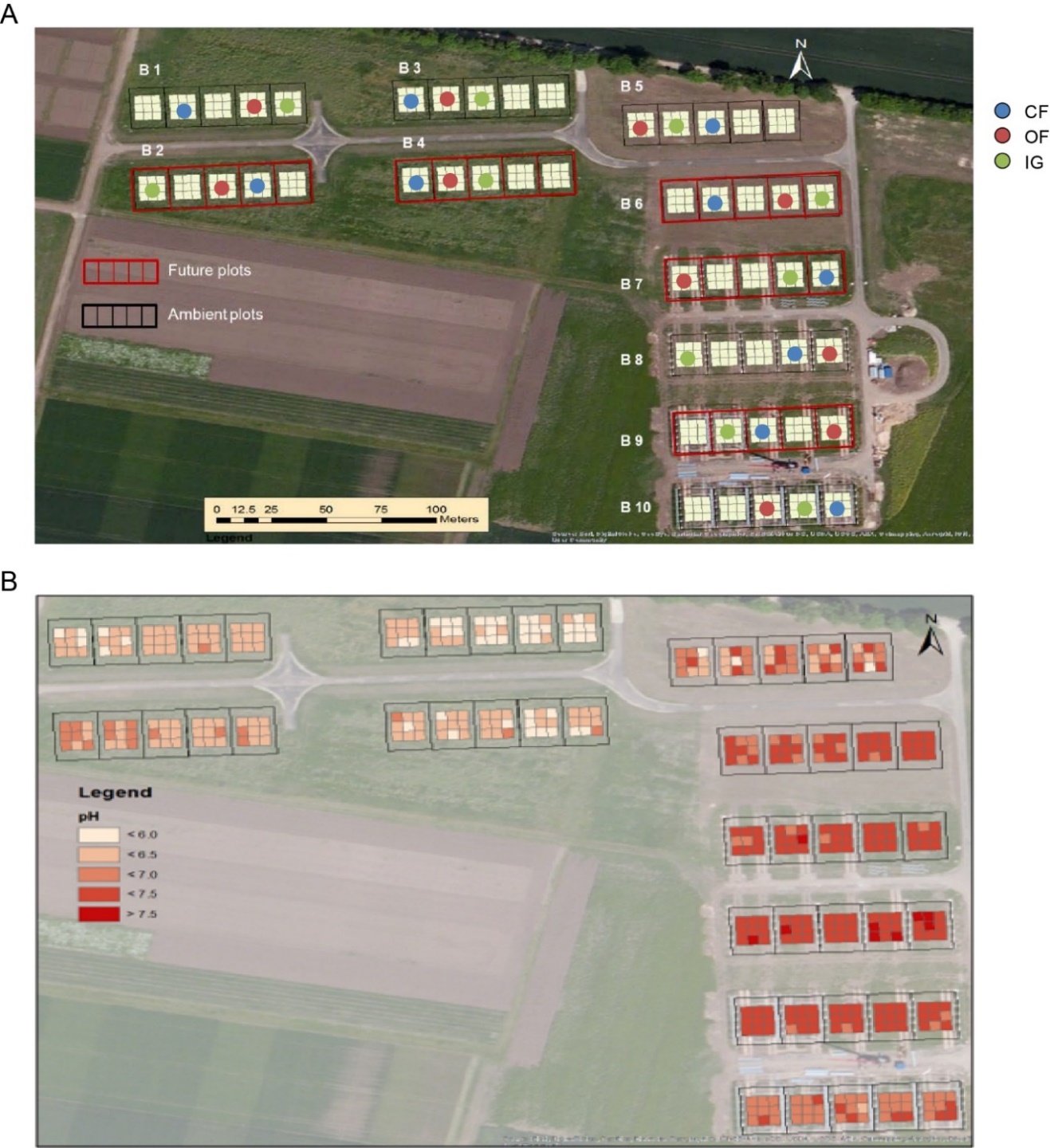


**Fig. S1:** **Layout of GCEF research station (A) and soil pH values of plots (B).**  Soil pH values were collected in the sampling campaign of June 2013. GCEF comprises 10 main plots (B1-B10), and each consisting of five sub-plots. CF: conventional farming; OF: organic farming; IG: intensive grassland. For more details on the GCEF research station, please see previous studies [1, 2]. Photo: UFZ.


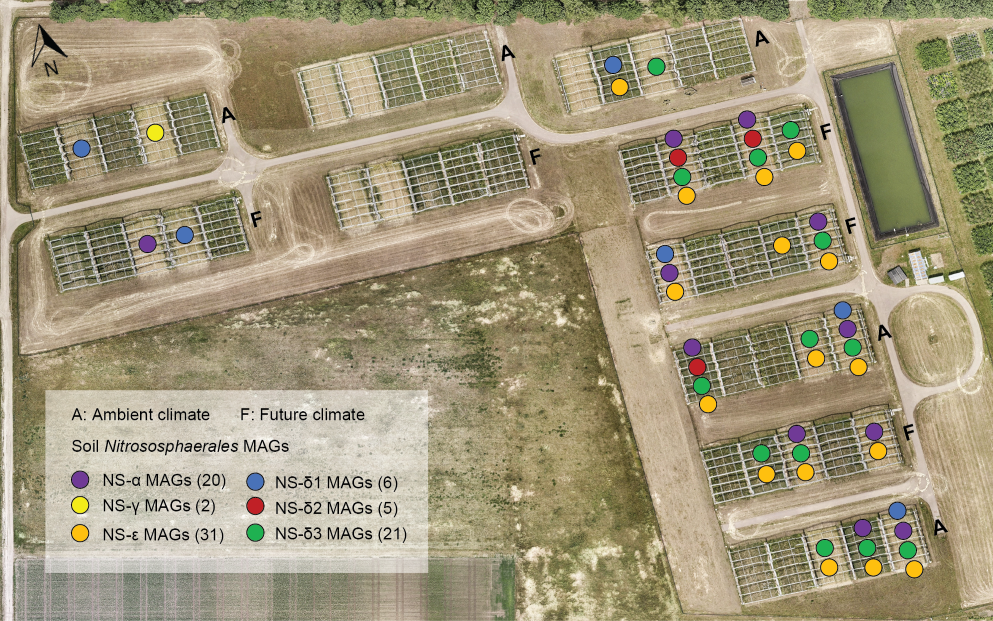


**Fig. S2: Layout of GCEF research station and plots from which 85 medium-quality AOA metagenome-assembled genomes (MAGs) were recovered.** Medium-quality MAGs were greater than 80% completeness with less than 10% contamination. See Table S3 for detailed information on recovered AOA MAGs belong to the order *Nitrososphaerales* (NS). A: ambient climate; F: future climate. Photo: UFZ.


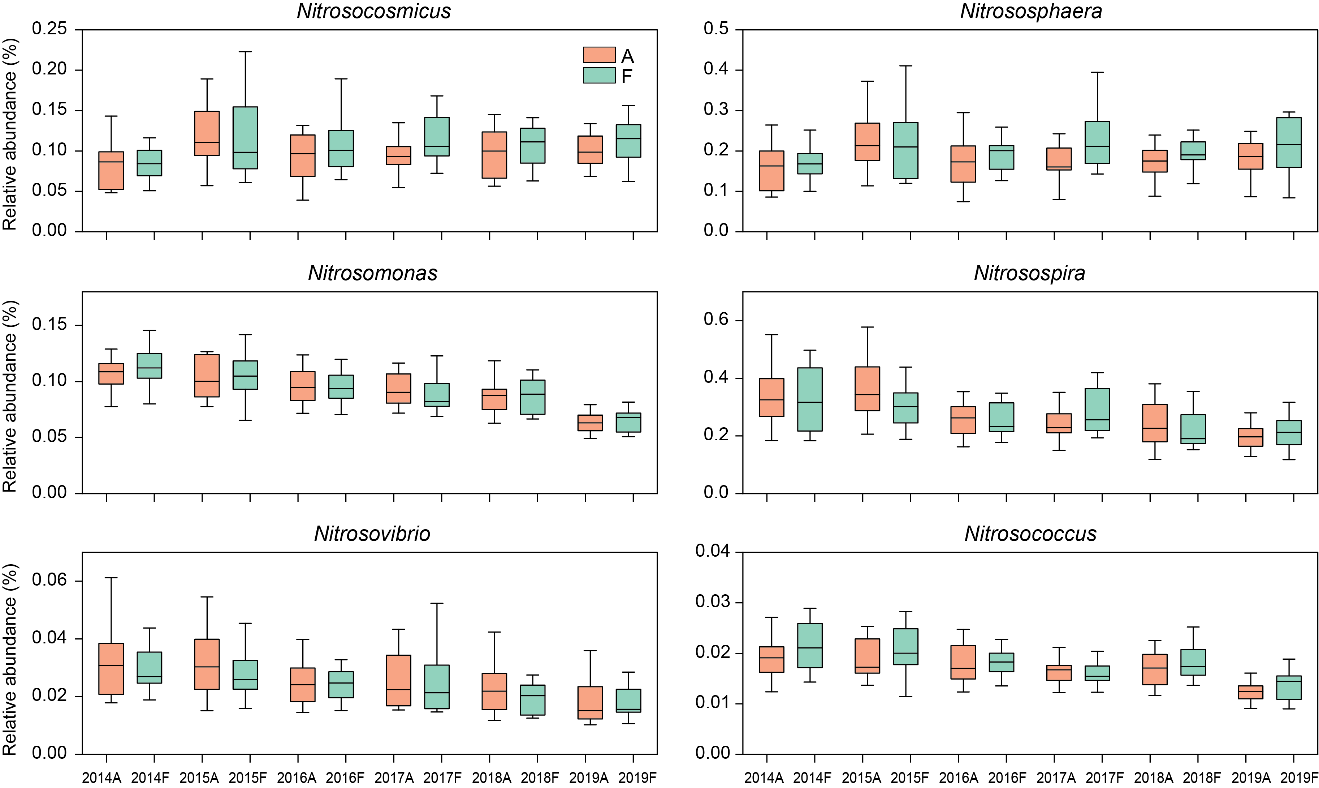


**Fig. S3:** **Relative abundance of soil ammonia-oxidizing archaea (AOA) and ammonia-oxidizing bacteria (AOB) across 10 plots at the GCEF during 2014-2019 summers.** The taxonomic profiles at the genus level were generated from the metagenomic FASTQ reads using Kaiju [1]. A, ambient climate; F, future climate.


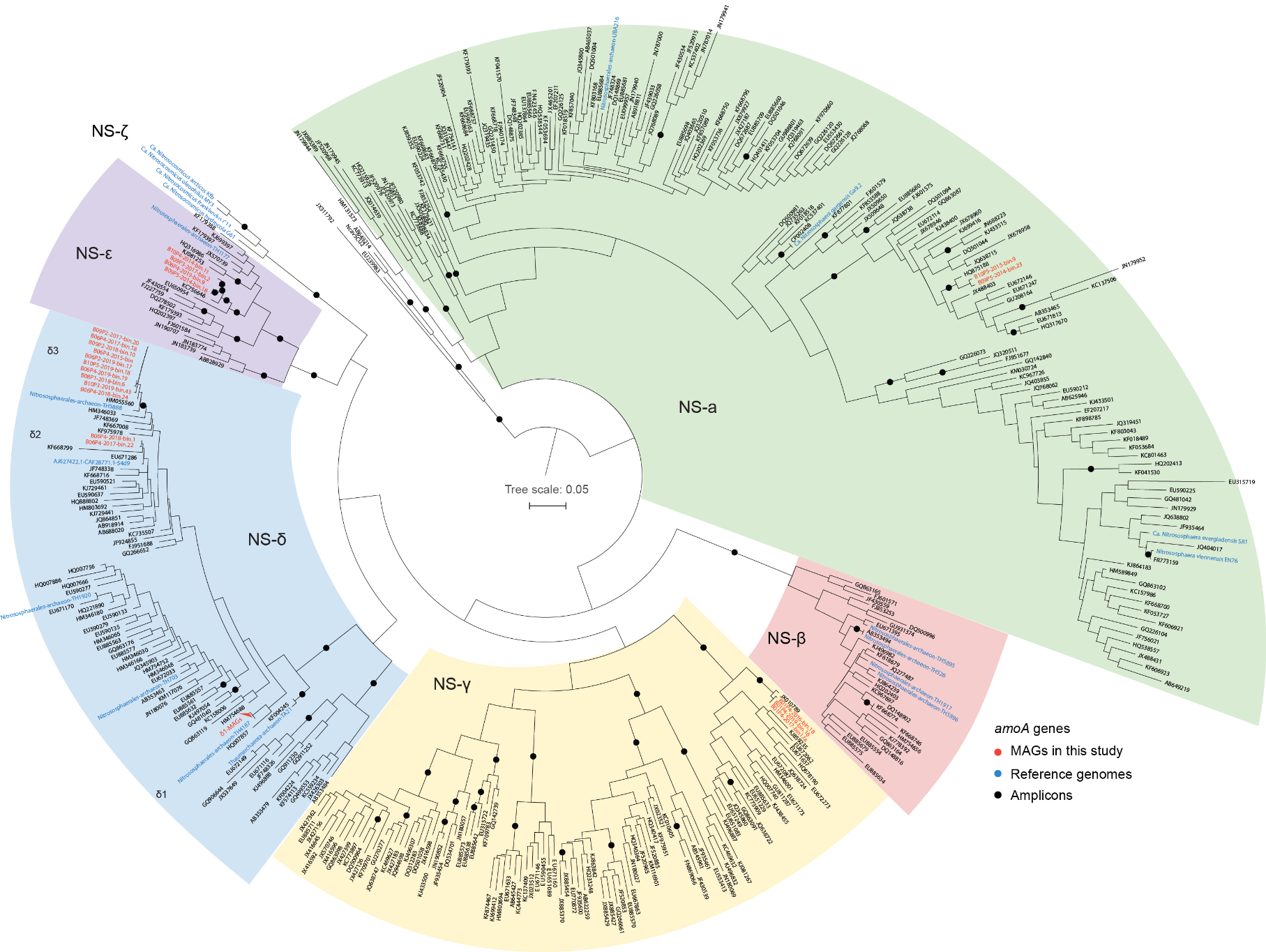


**Fig. S4:** **Phylogenetic tree of *amoA* genes identified in recovered AOA MAGs and reference genomes.** The tree was generated using maximum likelihood estimation with RAxML. Bootstrap values > 90 are shown. MAGs recovered in this study were marked in red. The representative *amoA* gene amplicons were downloaded from Alves et al. [3].


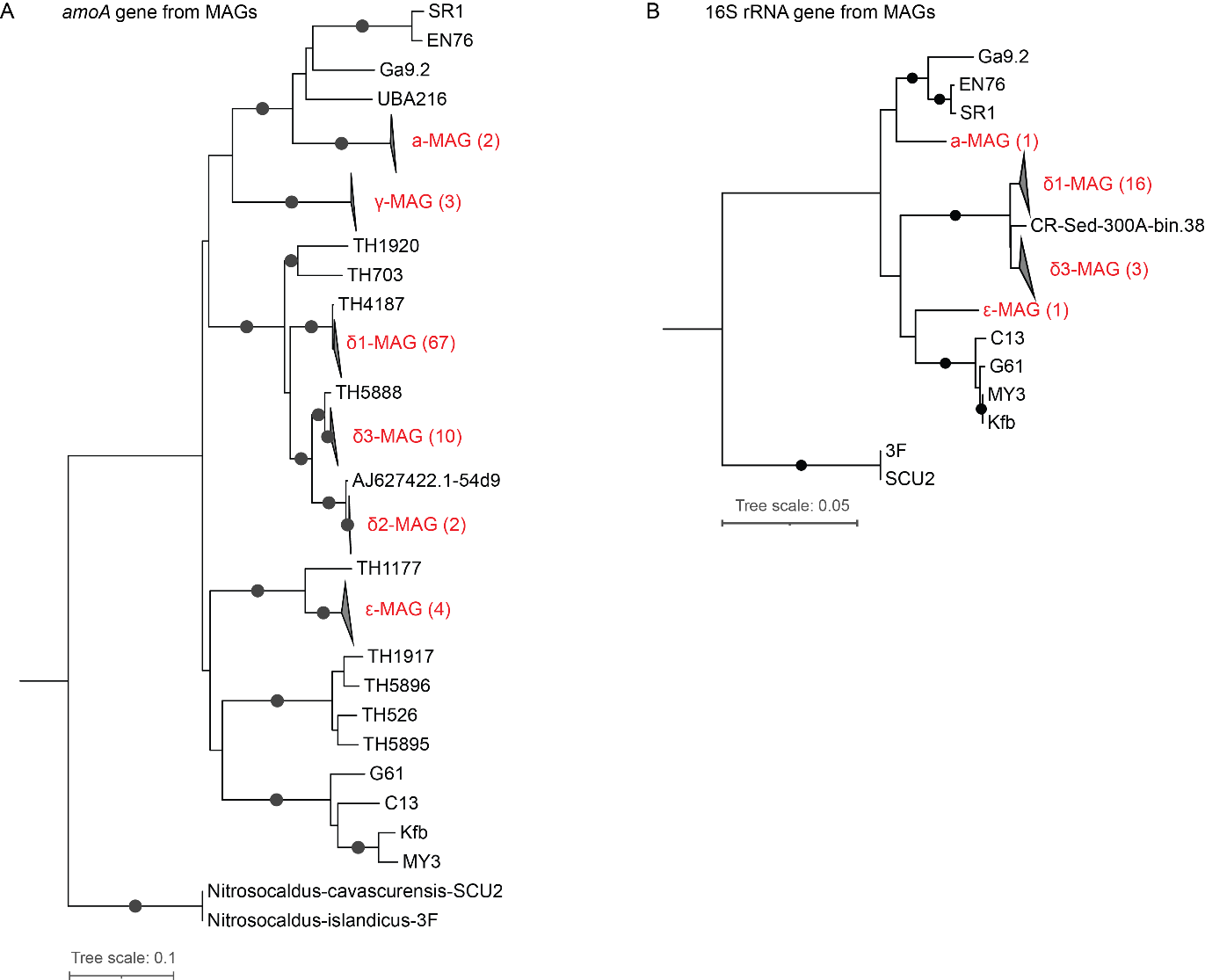


**Fig. S5:** **Phylogenetic trees of *amoA* (A) and 16S rRNA (B) genes identified in recovered AOA MAGs.** The tree was inferred from nucleotides using the neighbor-joining algorithm with the Kimura 2-parameter model (1000 bootstraps). MAGs indicated in red were assembled in this study. The number of 16S rRNA and *amoA* gene sequences in recovered MAGs was indicated in parentheses. The bootstraps larger than 90% were indicated by black dots. The trees were rooted with the *Ca*. Nitrosocaldales strains 3F and SCU2.


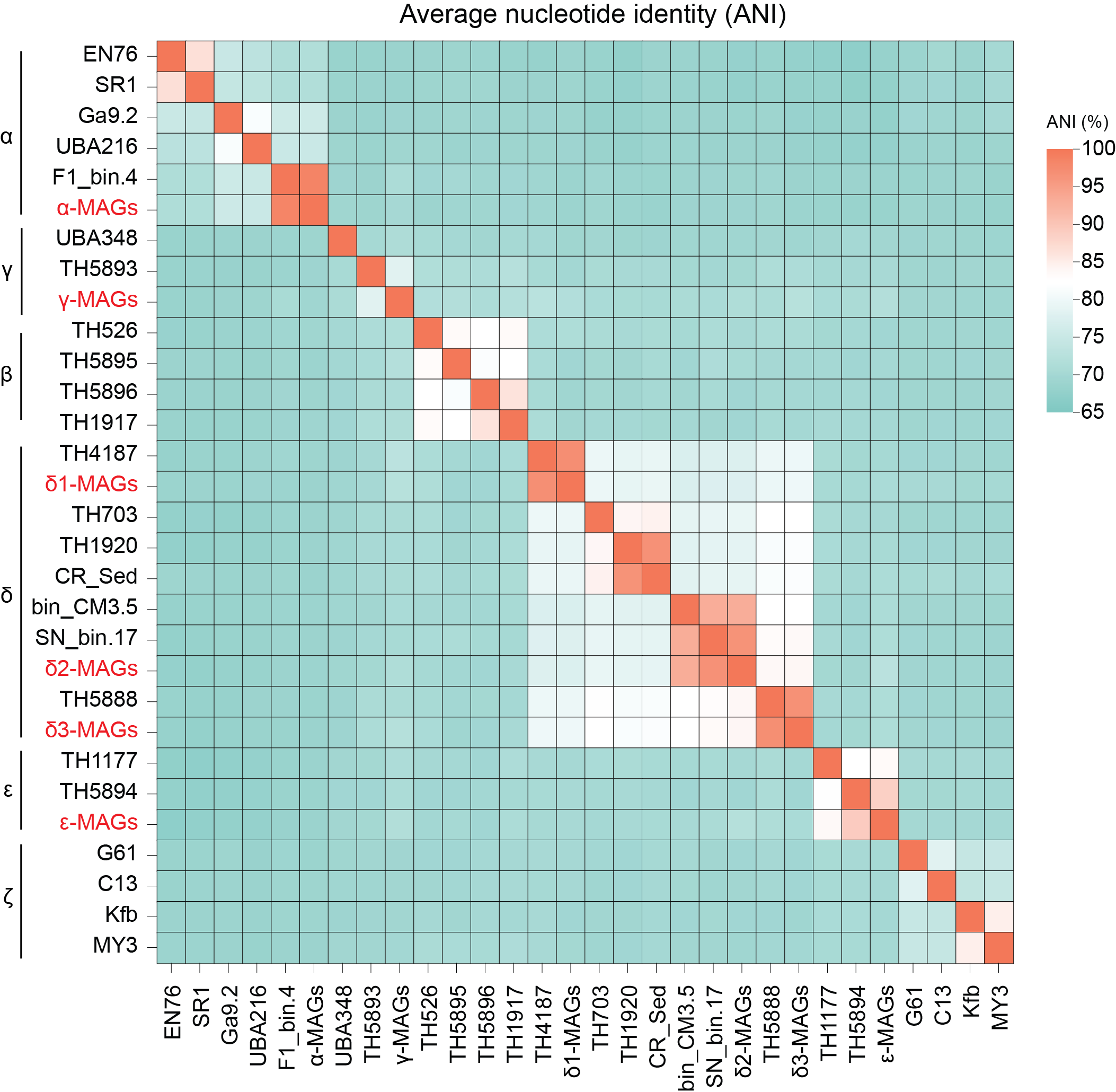


**Fig. S6:** **Average Nucleotide Identity (ANI) analysis of AOA genomes.** MAGs indicated in red were assembled in this study. See Table S5 for the pairwise ANI values.


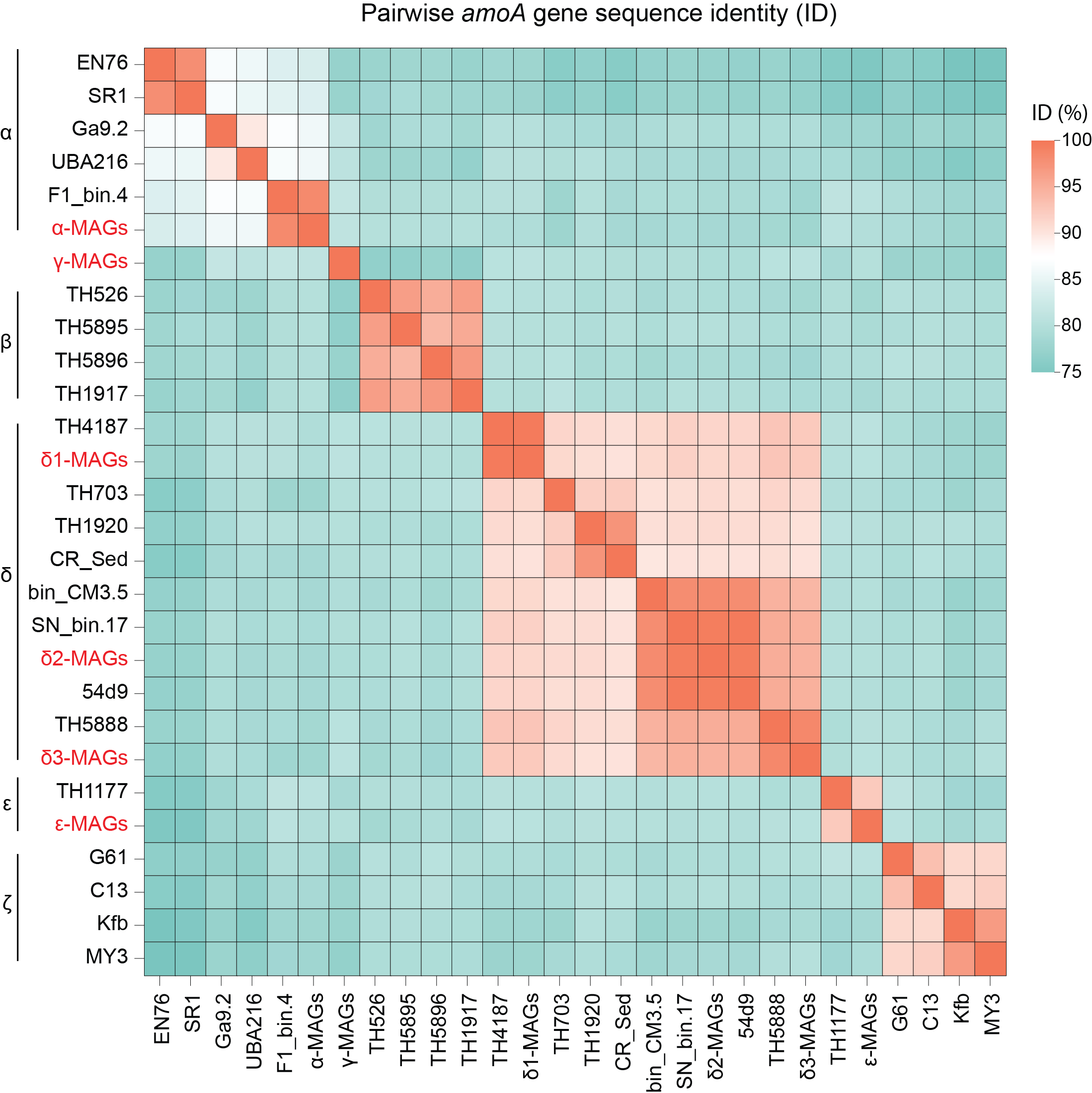


**Fig. S7: Pairwise identity of *amoA* genes derived from AOA genomes.** MAGs indicated in red were assembled in this study.


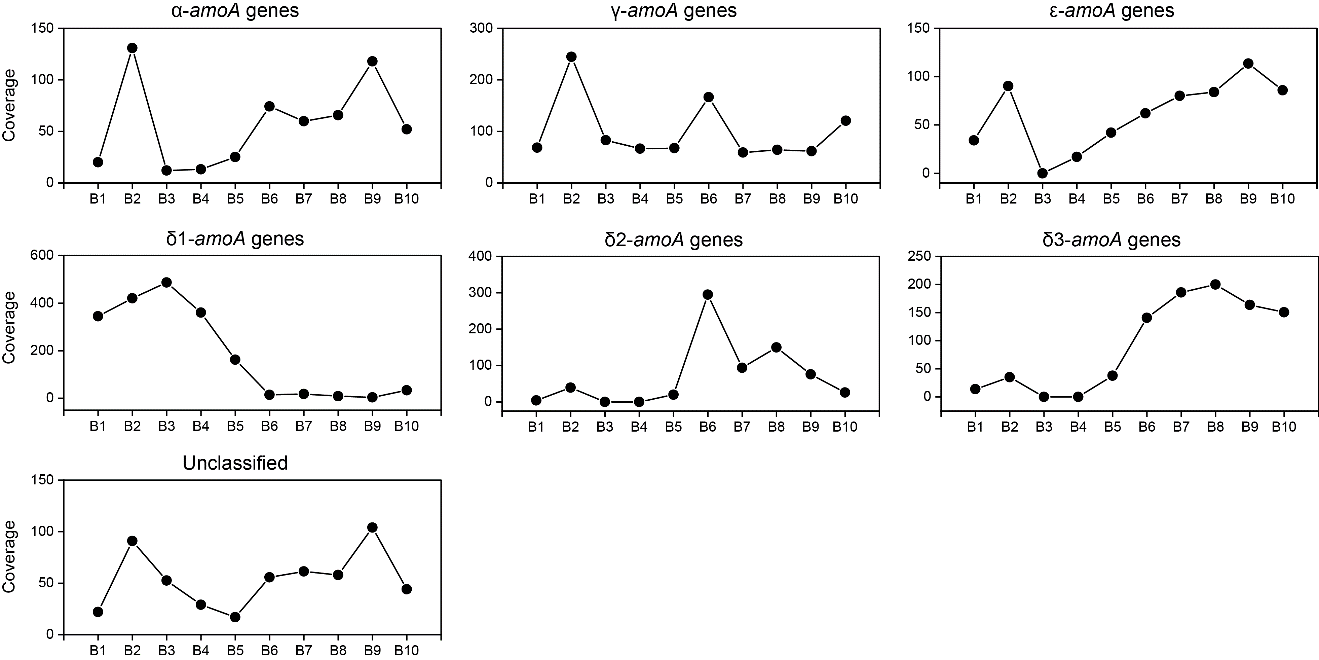


**Fig. S8: Metagenomic read coverage of contigs containing *amoA* genes affiliated with different NS clades across 10 plots at the GCEF during the 2014-2019 summers.** The coverage was calculated during the metagenome assembly using MEGAHIT within metaWRAP. A total of 659 *amoA* genes were retrieved from contigs across all samples, and 550 *amoA* genes were assigned to six NS clades with an identity of 96%. The remaining 109 *amoA* genes were classified as unknown.


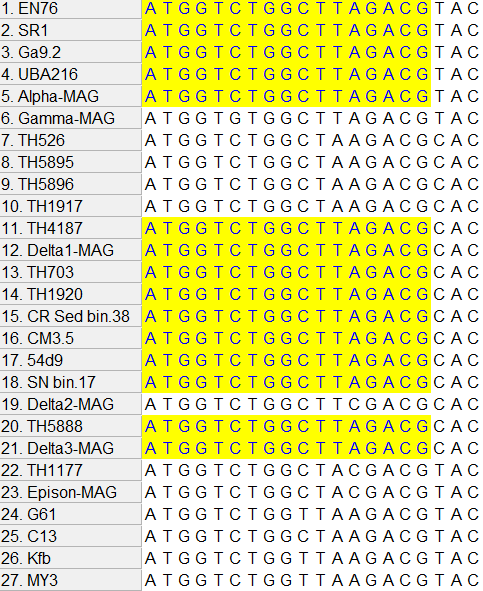
……………
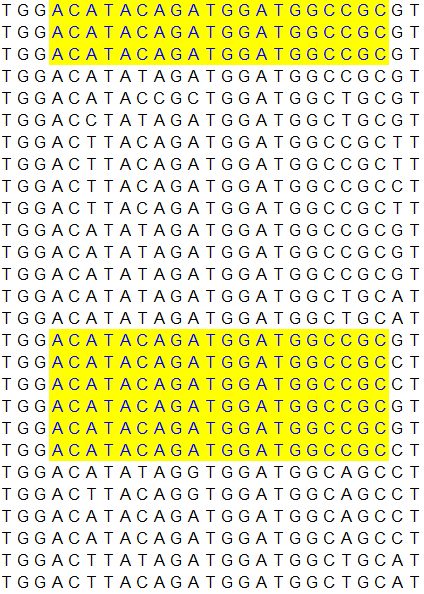


**Fig. S9: Primer-target regions of *amoA* genes derived from AOA genomes.** The primers Arch-amoAF (5’-STAATGGTCTGGCTTAGACG-3’) and Arch-amoAR (5’-GCGGCCATCCATCTGTATGT-3’) were often used for polymerase chain reaction (PCR) amplification targeting the archaeal *amoA* genes [4]. Sequences with yellow color indicates the forward and reverse primers.


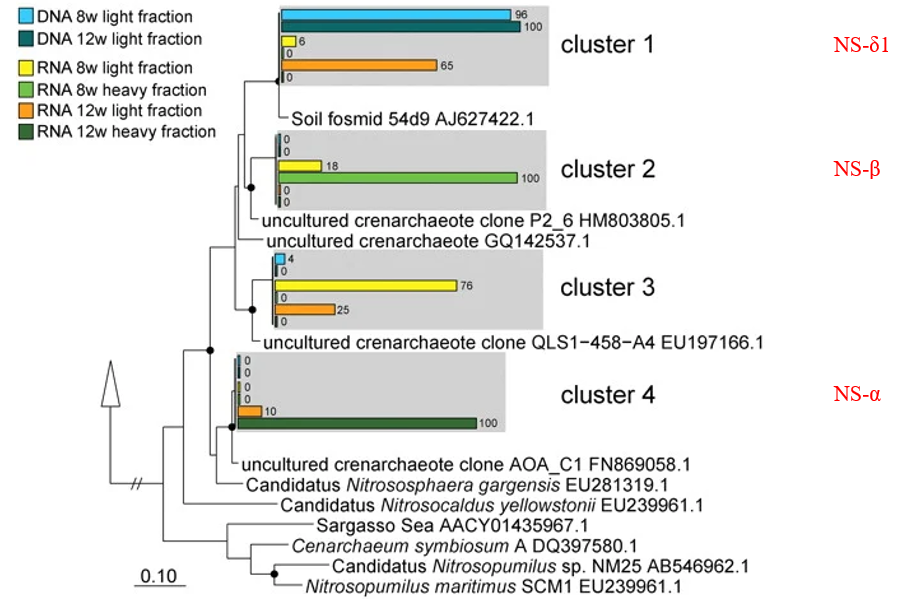


**Fig. S10:** **The *amoA* genes derived from recovered MAGs exhibit high similarity with sequences from a previous study.** See Table S12 for detailed BLASTN results of *amoA* genes in the recovered MAGs and previous study [5].


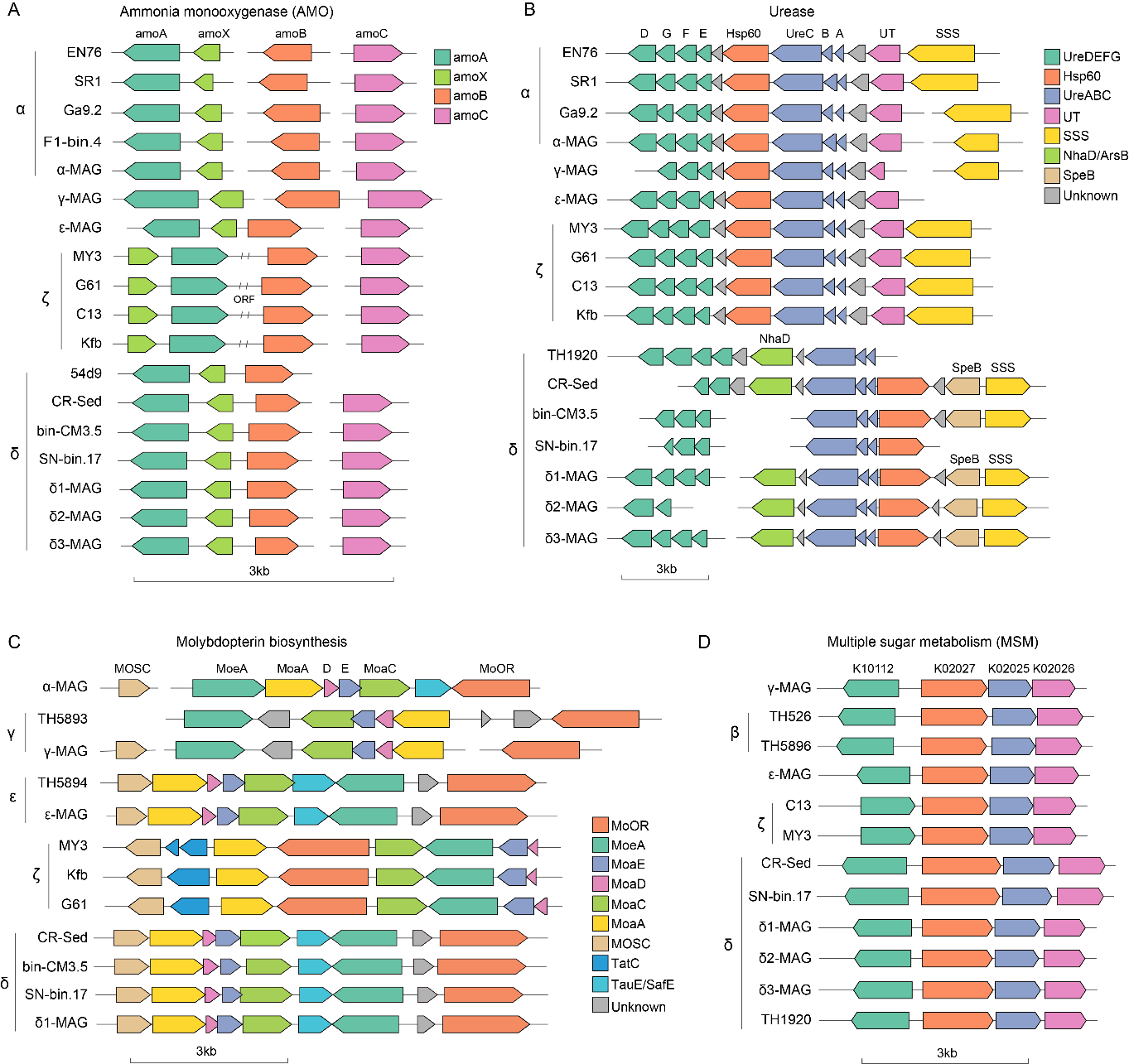


**Fig. S11:** **Representative gene clusters for ammonia monooxygenase (A), urease (B), molybdopterin biosynthesis (C), and multiple sugar transport system (D) in AOA genomes.** Open reading frames (ORFs) were represented by arrowed boxes. Map of the gene clusters was based on the reference genomes and recovered MAGs. The most intact ORFs were seleceted for the gene clusters in recovered MAGs. The colors of gene boxes indicate the gene families. Gray boxes correspond to genes with unknown functions.


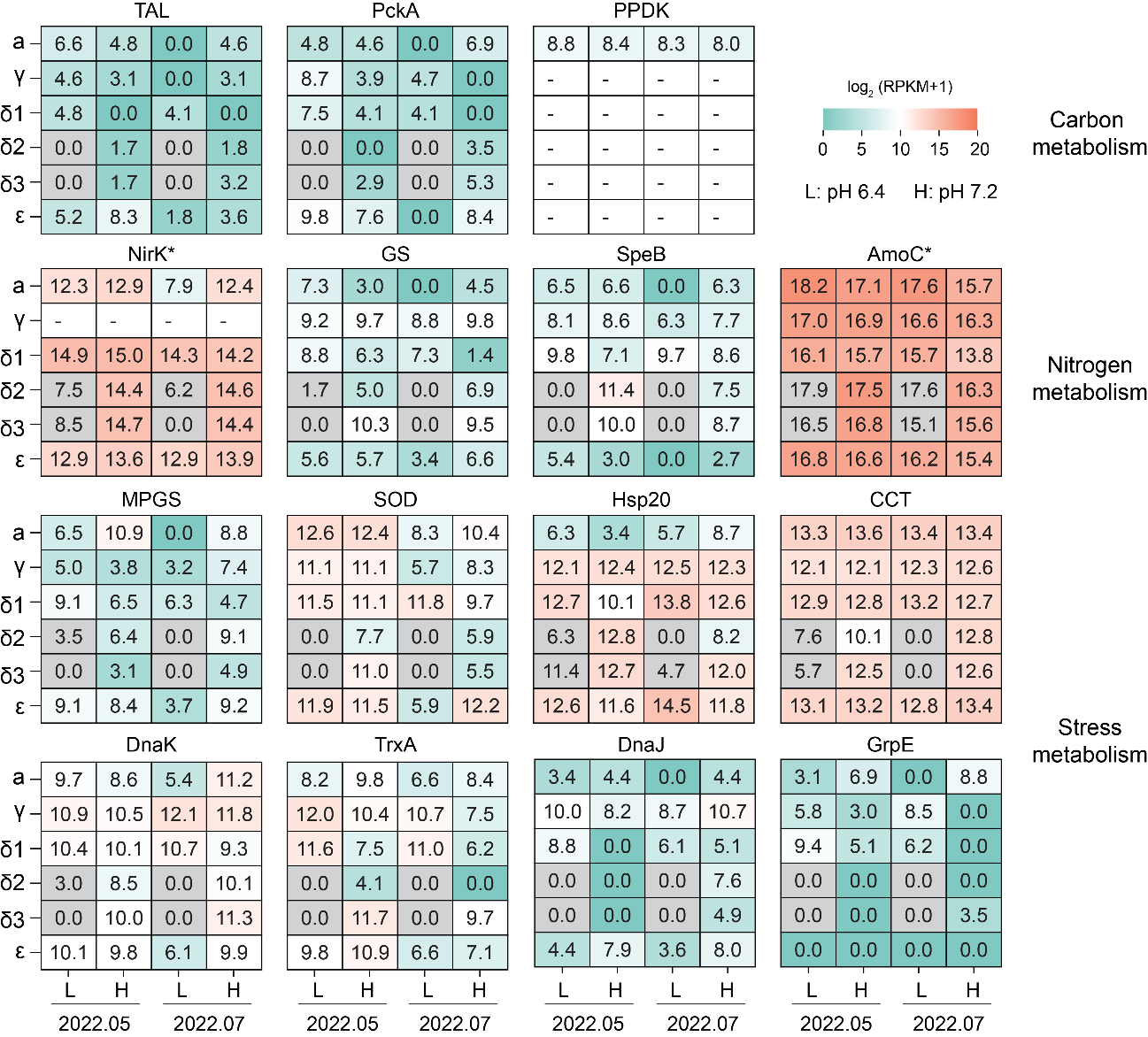


**Fig. S12: Expression of genes related to carbon, nitrogen and stress related metabolisms in recovered AOA MAGs.** Mean values were reported only if they were observed in at least two out of six replicates. Dashes represent the absence of the selected gene in MAGs. L and H denote groups of plots with mean soil pH values of 6.4 and 7.2, respectively. TAL, transaldolase; PckA, phosphoenolpyruvate carboxykinase; PPDK, pyruvate orthophosphate dikinase; NirK, nitrite reductase; GS, glutamine synthetase; SpeB, agmatinase; MPGS, mannosyl-3-phosphoglycerate synthase; SOD, superoxide dismutase; Hsp20, heat shock protein; TrxA, thioredoxin; CCT, archaeal chaperonin; Dnak/DnaJ, molecular chaperone; GrpE, molecular chaperone. *Only one ε-MAG contained the *nirK* gene, and none was detected in the γ-MAGs. *Only one α-MAG contained a partial (172 bp) *amoC* gene. A grey background indicates genes in MAGs that may lack sufficient coverage from metatranscriptome sequencing.


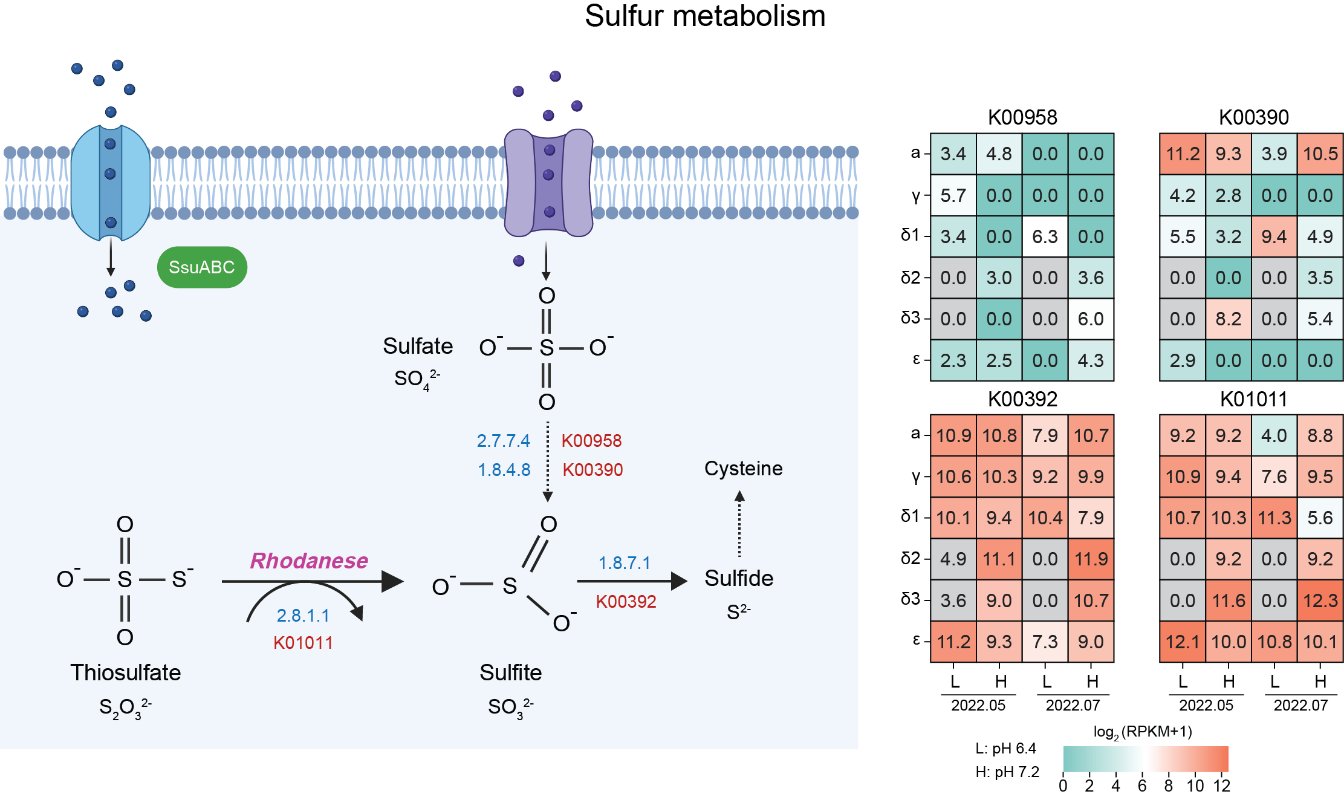


**Fig. S13: Expression of genes related to sulfur metabolism in recovered AOA MAGs.** Mean values were reported only if they were observed in at least two out of six replicates. L and H represent groups of plots with mean soil pH values of 6.4 and 7.2, respectively. A grey background indicates genes in MAGs that may lack sufficient coverage from metatranscriptome sequencing.


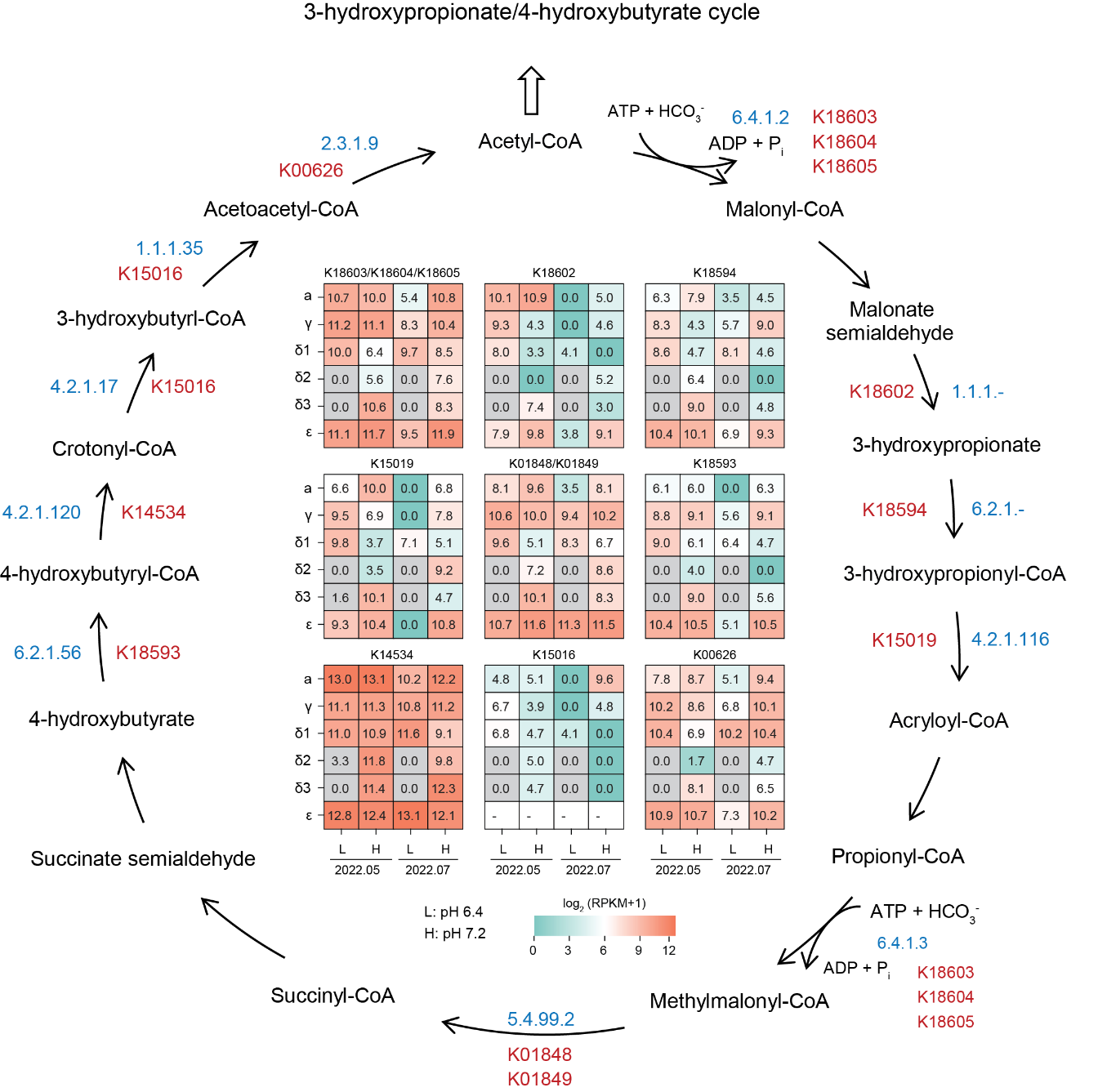


**Fig. S14: Expression of genes related to 3-hydroxypropionate/4-hydroxybutyrate cycle (3-HP/4-HB) in recovered AOA MAGs.** Mean values were reported only if they were observed in at least two out of six replicates. Dashes represent the absence of the selected gene in MAGs. L and H represent groups of plots with mean soil pH values of 6.4 and 7.2, respectively. A grey background indicates genes in MAGs that may lack sufficient coverage from metatranscriptome sequencing.


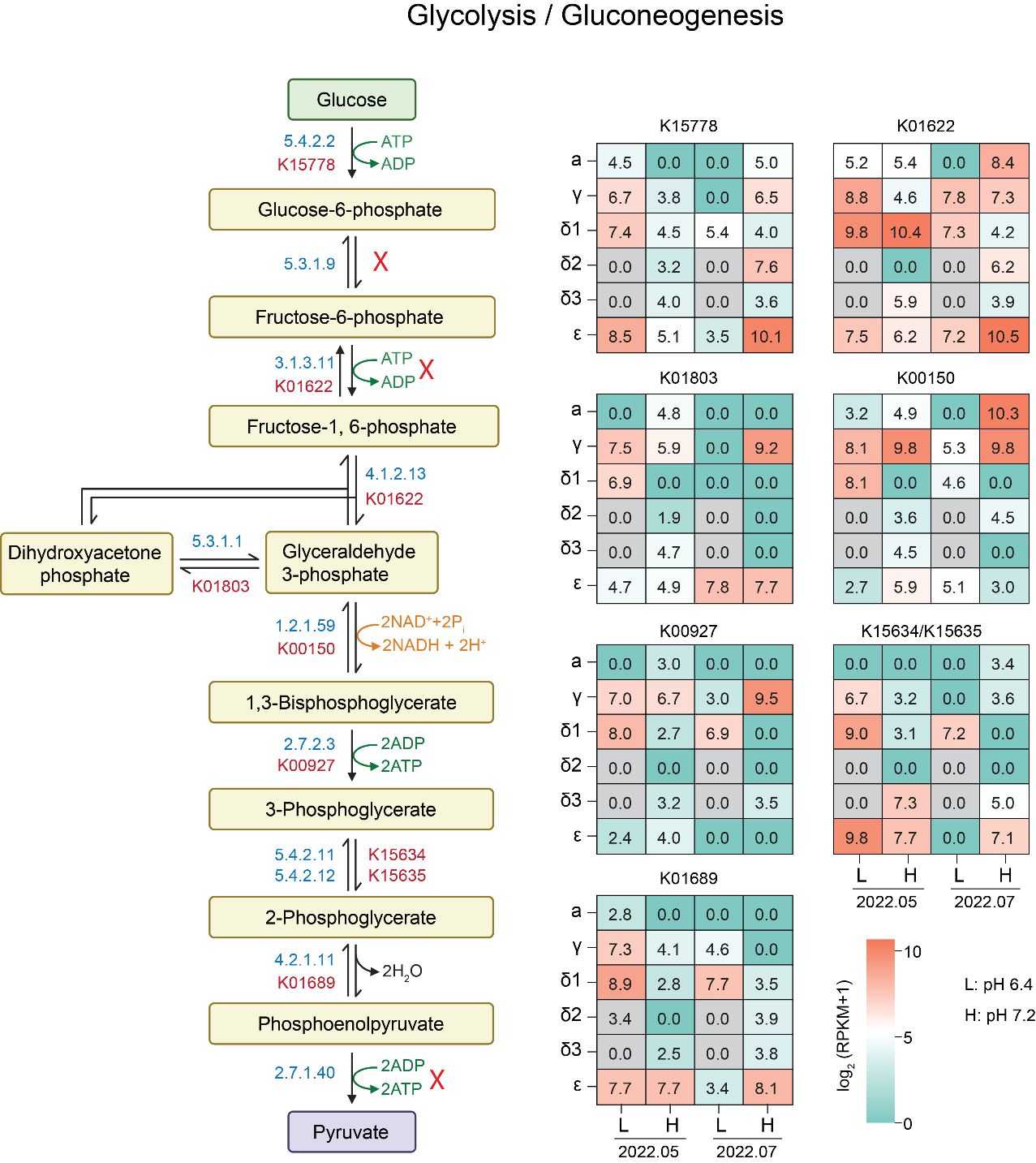


**Fig. S15: Expression of genes related to glycolysis and gluconeogenesis pathways in recovered AOA MAGs.** Mean values were reported only if they were observed in at least two out of six replicates. L and H represent groups of plots with mean soil pH values of 6.4 and 7.2, respectively. A grey background indicates genes in MAGs that may lack sufficient coverage from metatranscriptome sequencing.


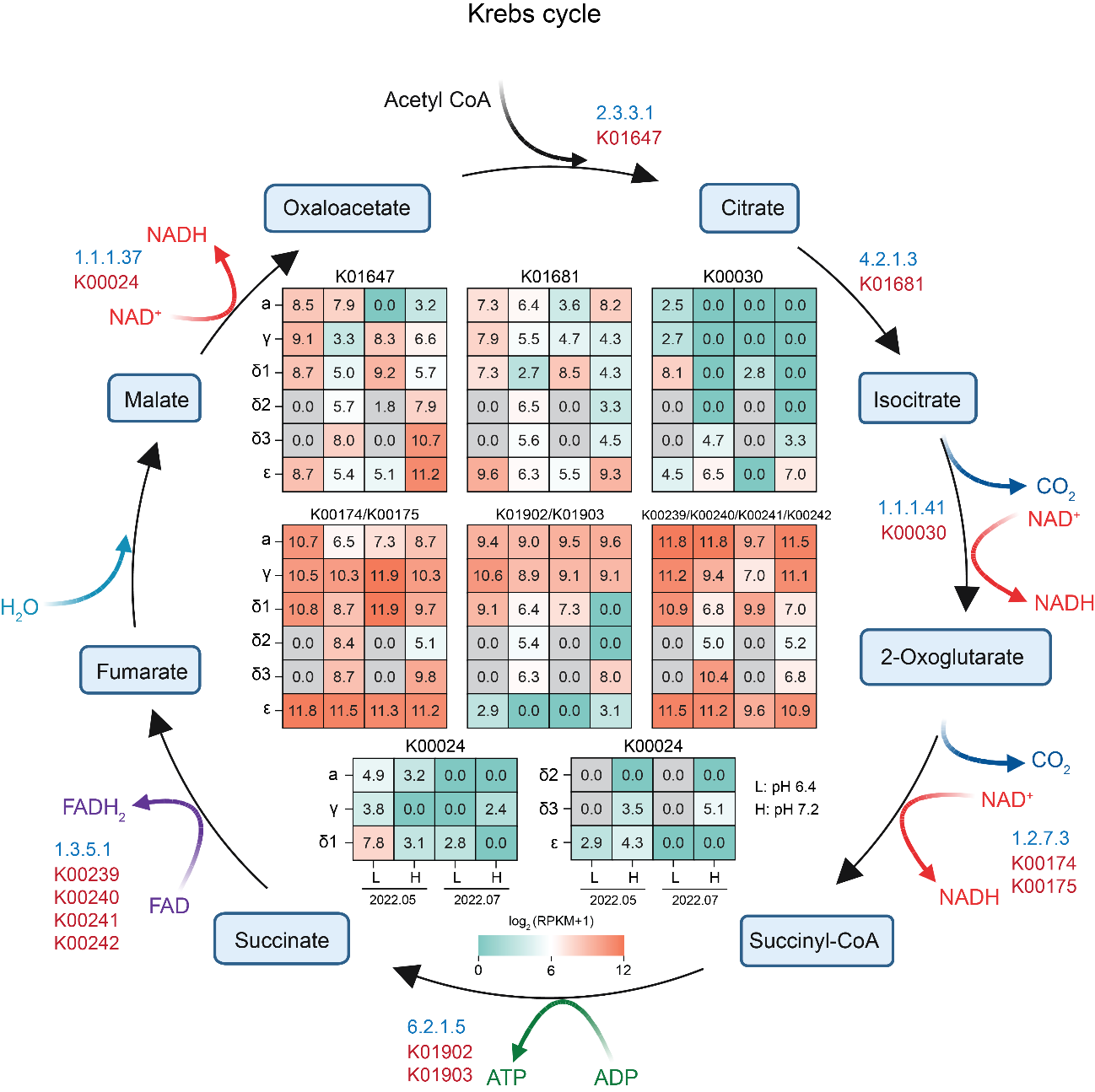


**Fig. S16: Expression of genes related to Krebs cycle in recovered AOA MAGs.** Mean values were reported only if they were observed in at least two out of six replicates. L and H represent groups of plots with mean soil pH values of 6.4 and 7.2, respectively. A grey background indicates genes in MAGs that may lack sufficient coverage from metatranscriptome sequencing.


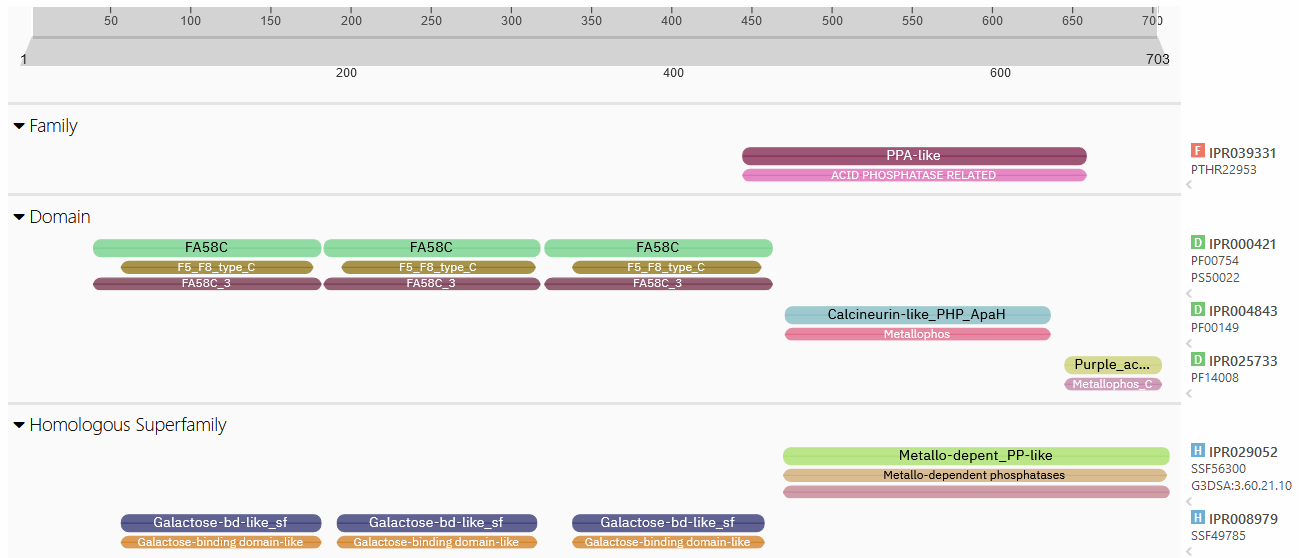


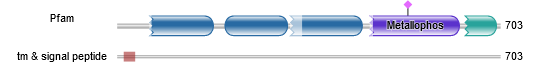


**Fig. S17:** **Functional annotation of 3CBM32 family in recovered NS-δ1 MAGs**. The annotation of the 3CBM32 family was based on InterProScan (http://www.ebi.ac.uk/interpro/) and HMMER (https://www.ebi.ac.uk/Tools/hmmer/search/hmmscan). See the amino acid sequence of 3CBM32 in recovered δ1-MAGs below.


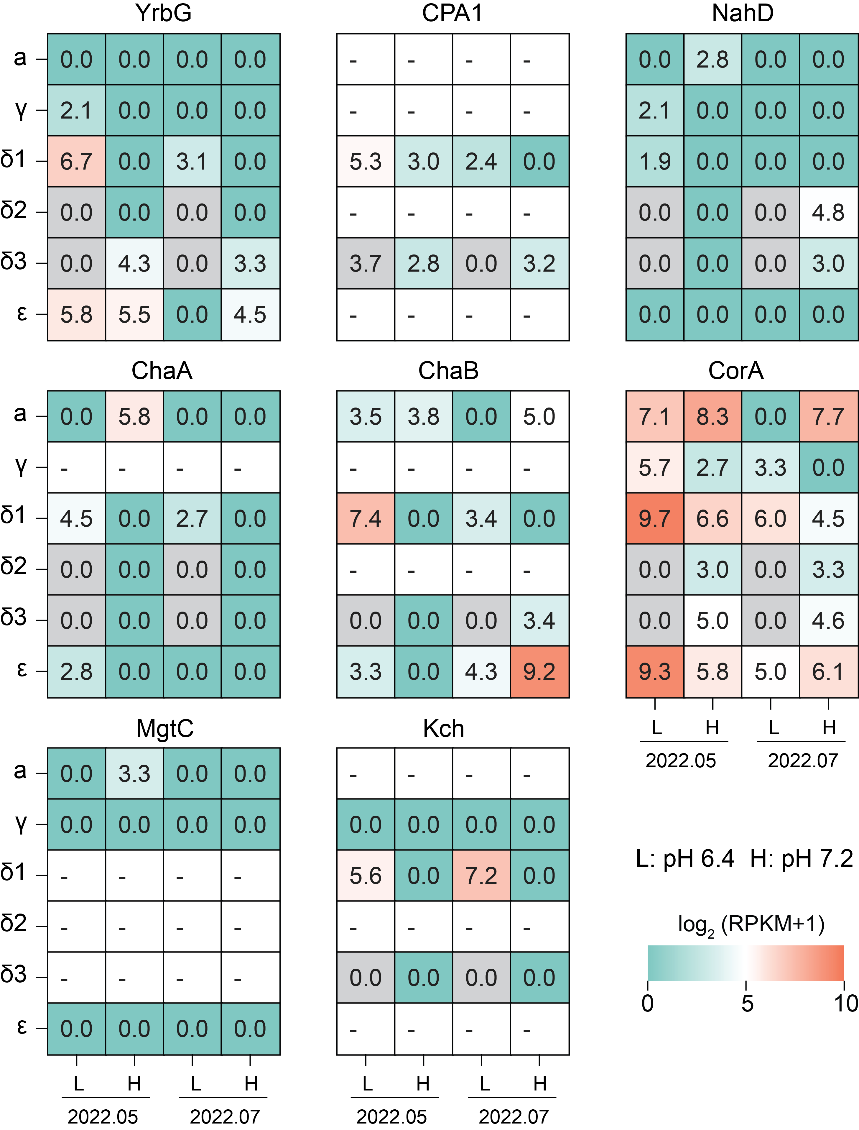


**Fig. S18: Expression of transporter-related genes in recovered AOA MAGs.** Metatranscriptomic Mean values were reported only if they were observed in at least two out of six replicates. YrbG, Ca^2+^/Na^+^ antiporter; CPA, cation/proton antiporter; Cha, Na^+^(Ca^2+^)/H^+^ antiporter; NahD, Na^+^/H^+^ antiporter; CorA/MgtC, mg^2+^ transport system; Kch, K^+^ transport system. Dashes represent the absence of the selected gene in MAGs. L and H represent groups of plots with mean soil pH values of 6.4 and 7.2, respectively. A grey background indicates genes in MAGs that may lack sufficient coverage from metatranscriptome sequencing.

**References**

1. Bei Q, Reitz T, Schnabel B, Eisenhauer N, Schädler M, Buscot F, et al. Extreme summers impact cropland and grassland soil microbiomes. ISME J. 2023;17:1589-1600.

2. Schädler M, Buscot F, Klotz S, Reitz T, Durka W, Bumberger J, et al. Investigating the consequences of climate change under different land‐use regimes: a novel experimental infrastructure. Ecosphere. 2019;10:e02635.

3. Alves RJE, Minh BQ, Urich T, von Haeseler A, Schleper C. Unifying the global phylogeny and environmental distribution of ammonia-oxidising archaea based on *amoA* genes. Nat Commun. 2018;9:1-17.

4. Francis CA, Roberts KJ, Beman JM, Santoro AE, Oakley BB. Ubiquity and diversity of ammonia-oxidizing archaea in water columns and sediments of the ocean. Proc Natl Acad Sci USA. 2005;102:14683-14688.

5. Pratscher J, Dumont MG, Conrad R. Ammonia oxidation coupled to CO_2_ fixation by archaea and bacteria in an agricultural soil. Proc Natl Acad Sci USA. 2011;108:4170-4175.

**Representative *amoA* sequences in recovered AOA MAGs**

>Alpha-MAG

ATGGTCTGGCTTAGACGTACAACGCACTACTTGTTCATAGTGGTGGTTGCTGTCAACAGCACGCTGCTAACTATCAACGCAGGAGACTATATCTTCTACACAGACTGGGCATGGACATCATTTGTCGTATTTTCGATCTCTCAATCTACAATGCTTGCTGTGGGAGCGATATACTATATGCTGTTCACAGGAGTTCCAGGAACTGCCACATATTATGCTACAATCATGACTATCTACACTTGGGTCGCCAAGGGTGCATGGTTTGCATTGGGCTATCCTTATGACTTCATTACGGTACCTGTGTGGATACCATCGGCAATGCTGTTGGACCTAACATACTGGGCTACGAGAAGGAACAAACACGCTGCTATAATCATAGGCGGAACGCTGATTGGCCTGTCATTGCCGCTGTTCAACATGGTCAACCTGCTGCTCATAAGGGATCCACTTGAAGTGGCATTCAAGTACCCGAGACCTACACTGCCATCATATATGACTCCTATCGAACCTCAGGTCGGTAAGTTCTACAATAGTCCTGTGGCCTTGGGAGCAGGCGCGGGAGCAGTGCTGACGGTTCCGATAGCTGCGTTAGGTGCAAAGCTCAACACGTGGACATACCGCTGGATGGC

>Delta1-MAG

ATGGTCTGGCTTAGACGCACTACACACTATCTATTCATAGTTGTAGTTGCTGTAAATAGTACCTTATTGACAATCAACGCAGGAGATTACATCTTCTACACTGATTGGGCGTGGACATCATTCGTAGTGTTCTCAATATCCCAATCAACCATGCTTGTAGTTGGTGCAATCTATTACATGCTATTCACCGGAGTACCAGGGACTGCAACATATTATGCAACAATCATGACTATCTATACATGGGTAGCCAAAGGAGCTTGGTTTGCACTAGGATATCCATATGACTTCATAGTAACACCCGTGTGGATACCTTCAGCAATGCTGCTAGATTTGACATATTGGGCAACAAGAAGGAATAAACACGCTGCCATTATTATTGGCGGAACGCTGGTTGGTCTTTCATTGCCAATATTCAATATGATCAATCTGTTGTTAGTCAGAGATCCACTGGAGATGGCATTCAAGTATCCTCGTCCAACATTGCCTCCGTATATGACGCCAATTGAGCCTCAGGTCGGAAAGTTCTACAATAGTCCCGTGGCGCTAGGATCGGGAGCTGGAGCTGTGCTGAGTGTTCCGATAGCTGCACTGGGTGCGAAACTCAATACTTGGACATATAGATGGATGGC

>Delta2-MAG (54d9-like)

ATGGTCTGGCTTAGACGCACTACACACTATCTATTCATAGTTGTAGTTGCTGTAAATAGTACATTATTGACAATCAACGCAGGAGACTACATATTCTATACAGATTGGGCATGGACATCATTTGTTGTATTCTCAGTATCCCAATCAACAATGCTTGTAGTGGGAGCAATCTATTACATGCTATTCACCGGTGTACCAGGGACTGCAACATATTATGCAACAATCATGACTATCTATACATGGGTAGCCAAAGGAGCATGGTTTGCACTAGGATATCCATACGACTTCATCGTAACTCCAGTTTGGATACCTTCAGCAATGCTGTTAGATTTGACATATTGGGCAACAAGAAGGAATAAACATGCTGCCATTATAATTGGCGGAACATTGGTTGGACTTTCATTGCCAATATTCAACATGATAAATCTACTGCTGGTCAGAGACCCTCTAGAGATGGCATTCAAGTATCCTCGTCCAACATTGCCTCCATACATGACACCAATTGAGCCTCAGGTCGGTAAATTCTACAATAGTCCCGTGGCGCTAGGATCCGGGGCGGGAGCTGTACTTAGCGTTCCAATCGCTGCATTGGGCGCAAAACTAAATACCTGGACATACAGATGGATGGC

>Delta3-MAG

ATGGTCTGGCTTCGACGCACTACACACTATCTATTCATAGTTGTAGTTGCTGTAAATAGTACATTATTGACAATCAACGCAGGAGACTACATTTTCTATACAGATTGGGCATGGACATCATTCGTAGTATTCTCAATATCCCAATCAACAATGCTTGTAGTTGGTGCAATCTATTACATGCTCTTCACCGGTGTACCAGGGACTGCAACATATTATGCAACAATCATGACTATCTATACATGGGTAGCCAAAGGAGCTTGGTTTGCATTAGGATATCCGTACGACTTCGTGGTGACTCCAGTCTGGATACCTTCAGCAATGCTATTAGATTTGACATATTGGGCAACAAGAAGGAATAAGCATGCTGCCATTATAATTGGCGGTACATTGGTAGGACTTTCATTGCCAATATTCAATATGATAAATCTACTGCTGGTCAGAGATCCTCTAGAGATGGCATTCAAGTATCCTCGTCCAACATTGCCTCCATACATGACACCAATTGAGCCTCAGGTCGGAAAGTTCTACAATAGTCCCGTGGCGCTAGGATCGGGAGCTGCGGCTGTGCTGAGCGTTCCAATCGCTGCATTGGGCGCAAAACTCAATACTTGGACATACAGATGGATGGC

>Epsilon-MAG

ATGGTCTGGCTACGACGTACAACGCACTATCTATTCATAGTGGTCGTTGCTGTTAACAGTACATTATTGACTATCAATGCTGGTGACTATATCTTCTATACTGATTGGGCTTGGACTTCATTTGTAGTATTCTCTATTTCACAATCTACTATGCTTGTGGTTGGAGCAATTTATTATATGCTCTTCACAGGAGTTCCAGGTACGGCGACATATTACGCAACAATTATGACAATATATACTTGGGTTGCAAAAGGTGCATGGTTTTCTTTAGGATATCCCTATGACTTTGTAGTAGTACCAGTTTGGATACCTTCTGCAATGTTATTAGACCTATCATATTGGGCTACTCGACGAAACAAGCATGCAGCCATTCTAATAGGTGGAACACTGGTTGGAATGTCATTGCCTGTATTCAATATGATAAACCTGTTGCTAGTTAAGGATCCGCTAGAAATGGCATTCAAATATCCTAGACCTACATTGCCCCCGTATATGACGCCTATAGAACCTCAGGTCGGTAAGTTCTACAACAGTCCTGTAGCCTTAGGAGCAGGTGCAGGTGCAGTGCTTTGTGTGCCAATAGCAGCACTAGGCGCGAAGCTGAATACTTGGACTTACAGGTGGATGGC

>Gamma-MAG

ATGGTGTGGCTTAGACGTACAACGCACTATCTCTTCATAGTCGTAGTTGCTGTAAATAGTACATTGCTGACGATTAACGCGGGAGATTACATATTCTACACGGACTGGGCCTGGACCTCATTTGTAGTATTTTCCATCTCACAATCTACTATGCTTACTGTGGGTGCAATTTACTACATGCTCTTCACAGGAGTTCCAGGAACTGCAACCTATTATGCAACCATAATGACAATTTACACCTGGGTAGCAAAAGGTGCATGGTTTGCATTAGGATACCCGTATGATTTCATAGCAGTTCCCGTATGGATACCCTCAGCGATGTTGTTAGATCTCACGTACTGGGCTACTAGACGCAATAAGCACGCTGCAATTATCATCGGCGGAACTTTGGTTGGCTTGTCGCTGCCTATGTTCAATATGATAAACTTGCTTCTGGTGCGAGATCCGCTTGAGACAGCATTCAAGTATCCAAGACCTACATTGCCTCCATATATGACCCCAATTGAACCCCAGGTCGGAAAGTTCTACAATAGTCCTGTTGCCTTGGGATCAGGAGCATCCGCAGTTCTGACTGTGCCAATTGCGGCATTGGGTGCCAAGCTCAACACGTGGACCTATAGATGGATGGC

**Representative GH5 sequences in recovered AOA MAGs**

>Delta1-GH5-1

MKLSPFGILFLFLLILNVASISSLNPNLNLNLNLNLNLNPIYAQETNSQQLIGVNMLGYYTSLPQTRDFKNPFPDNYYDQSFKIIKDGGMNHVRYVYYWESYVKNPTAFINELKFAATIADKYGLKIIYDNHQFHTSSWLNPQRGTGFPNFLFQNNSEYAYGSGGGPKYPSAVAWWTHWWNREITDVNGTDGWTLQSQFLKKVVSTVDSHPSTVGYEILSEPQVHSVDQWDKVGKYNTFLADELRKVTKKDIVYSMSIPVDLKSNIGVNATNLAKMTPANKTNTIFKFSIYGLPSPGSYQEERLNMFIAAGNLSGVPVYIGEWNNVDRDKVINEEGDFVYEINPEESDITPADTTAILKKFNEIDPYGWAFWYWNFRPHRVENFNLVTSDPSGKLIPTKYFDILKNAVQSVNSGKGSK

>Delta1-GH5-2

MSSFRQNTPVLFGLSLVMSLFMTNGIIAASAQPSAADDTLVGVAMKGAYVDQKQNRPDTALPPATYFDESFKLLKAAGLNHARFLFYWEAYEKNPQAFMNEIEQVANIADKYGIKVIYDNHQWHTSSWLEKRGTGFPSALFENNSQLYPMNSGGKEGEPVAKLWWSNFWDGSVKDGQGKDAWTLLADFWKKVVTKVDGHPSTFGYEILSEPHVESSDQWEKIGNFNSFITDELRTLTNKTIVYSMNVPVDLGGPIELTPENLAKMAPSNKDNVWFKISVYGIPDRDKYQKERWDMFLKTRELTGDPLYIGEWNNVVRTQVNGVFQLDPAKSGLDQQTTDTMLETFKDSNITASSFWKWDYQDAPIASFNLILHNATQAPTDQNATQTPADQNSTLTPTEYYTYLKNSVTKIFPNLNPAN

>Delta1-GH5-3

MLIFILNWMQTSNLTKYLISFRIALFVAIFSLLLTNSFVLVSHGVETNQIFFGVNMKGYYTSMPQSRTIGSIMPPNYFDHSFKLISDAGMNHVRFVFYWEAYVKDPTNFMLELQSVAQAADKHNINVIYDNHQFHTSSWFNPRKGTGFPSLLFENNPSYIAGNGGGPKYAPAQLWWTDWWNRAVKDANGTDGWTLQAEFLEKIVNTLDSHKSTLGYEILSEPQVHNADQWEKIGQYNTFIVNELRKYTDKVIAYSMNIPVDLKSPINLTPENLAKMKPQNSTNVVFKISIYGLPTGSYQQQRLTTFLQASNMTGVPLYIGEWNNVLREQTINEEGATVFEINPFESDINQQEANQFVKIFKDLGIWGLAYWKWDYVLTQTPNFNLISISDNGDIVTNKYFKQLKIALDSNYGSQPSQESQ

>Delta1-GH5-4

MILASITKIFFAFVLAAILISSTMLSSYASTSKLSASQPINGVAMKGAFVNMKQHVENWPLAPQNYIEDSLRMISGAGLNHVRFVFYWEAYERDPKAFINEIESIAKAGDKYGLKIIYDNHQWHTSSWLEERGTGFPWSLFQDSKYPRGGGGNTHDKAAQVFWKDWWNRSIKDKQGKDGWTLMSEYLKEIVLAVDDHSSTLGYEILSEPHVDNKNQWSKIGKFNSFITAELRNLTSKTILYSMNVPVEFNSHINVSPKNLAKMAPSSRENIAFKISIYGVPDGDGYQEKRFDMFLKTRDITEIPLYIGEWNNVVRTKEGGITKLNPDLSQLTKTDAKKILGALKKEKVWGTAFWRWDYQPVVTDNFNLVSNKSGKLVPTKYLDILKDTVEKVYGSSSSTSADFTTSVGTSSIDPGQQTNLIKALVKTGKFTEAEAKQFLSKISQNRTEDISTSNDTESSSNQGNPKEYRTLNDLVDDINNNVVDIEEIPLNTFQDSDAYKGADKQTQNCLDLAGKIGDNLGDREIVHCSDDANYFQNKLF

>Delta1-GH5-5

MTSSQPINGVVMKGAFVNMKQHDNGSPEAPQDYIDDSLKMISKAGLDHARFLFYWEAFERDPKAFMKEIESVAKAGDKYGVKIIYDNHQWHTSSWLEDKGTGFPWSLFEDSKYSKGGGGNTPDKGAQVFWKDWWDRSVKDNDGKDGWTLMAEYLKKIVLAVDNHSSTLGYEILSEPHVDNTDQWSKIGKFNSFITEELRDITSKTIVYSMNVPVDLNSNINISPENLAKMAPSSKQNIAFKISVYGVPDRDDYQKERFDLFLDTRDLTGVPLYIGEWNNVVRTKEGGVFKINPGASDFTNSNAGKILEAFKKEGIWGTAFWKWDYRDADTASFNLVNDEGGKLVPTKYFGILEDTVEKVYGSFDSVSTSDVSASGNTSTGGDDDTSTSDVSASGNTSTSDDDTSTSDVSASGNISTPDDGKETNLINKLVKTGKFTEAEAKQFVSKSMQNGPDVTSTSDNTQSSNTQSSNNQTNSNDQPSKANTNNPEKYDNFDDLVDDIKNHVVDIEDISLNAFQDSGAYQGADQETQDCIDLAAKIGDNLGDQEIVNCSEDPNFYRNEISSTGSNDDNNSDNNGN

**Representative CBM32 sequences in recovered AOA MAGs**

> Delta1-3CBM32

MINLLKLSSCNAWRGNQLAFCMSVVTFLVLIFTNTLVQSNNIYGQSDPFNSANSCSKLSVSGITASGADASNPPSHAIDQNINSRWSNLGLGSWIQLDLGKENVICSIGINWHRGNERINSFVIAISKDGKTFTNVFSGKSDGTSLTEQEYNTQSKIGRFVRVTVTANTQSNWISISELKVYGYKPISESCVNSPISQVTAASSQVGLPPSNLVDNNLNTIWSNYGIGSSIQIDLGTSKRICNLDIAWYKGSERQNNFVISTSQDGKSFKNVLSTASSGKSTSYEKYVFSDNLARYIKITVNGNTQNNYASIAEIRAQAPASSQPQVECVDGHIQNAKTSGSQTSFPGPSVLDDKLDTRWSNNGVGSWIQLDLGTSNKICDINIAWYKANERQNNFVISTSTDGIKFSNVFSSKSSGSTLGLEKYNIADTNARFVRITVNGNTQNSWASITEVSVKIFPISGSSNYYIGAAGDWGSARNNNWEETVDLMINSKINLALGLGDYSYGSVSDFEPVVDTLNAAGIPFKGALGNHDSSSYASLFGQPSMLYAFDAGQARIILLNTEDSDSANTQFLENELKNTKQPWKIVAMHKPLYTSPSIHPEEKELAGKLQPLIDKYGVNLVLYGHNHNYERIKLPDKPTLYVQAGTAGESHYDIKGSRSGGGVEFKDDNDYGFVKLTINSDTLSGQFISHDGEILDSFGLSK

> Delta3-3CBM32-1

MKLGVSLVTFLVLIFSSTLVQPSHVYGQSDSFSSSNTCSKLPVNGITASGADPLQPPSHAIDQNVNTRWSNLGLGSWIQIDLGQENVICDVGINWHRGNERVNKFVISISKDGKTFTNVYSGKSDGTSLTEQKYDLQSKVGRFIRVMVDGNTQSNWISISEIKIYGYKAALSETCVKSQVPQATAAAASSQIGFPSSNVVDNNLNTIWSNYGVGSSIQLDLGSSKIICSLDIAWHKGNERQNNFVVSTSQDGKSYKTVLSTISSGKSLSYEKYNIADTNARYIRITVNGNTQNNYASIADIRAQISSSGQSQVQCVDGHIQNANTSASQTGFPGTNVLDNNLDTRWSNNGVGSWIQLDLGISNKVCDISIAWYKGNERQNNFVISTSNDGIKFSNVLSSKSSGSTLNLEKYDFTDINARYIRITVNGNTLNTYASITEVSLNTVSISGSSNYYIGAAGDWGSARNDNWEQTVDLMIDNKINLALGLGDYSYGSVEEFEPVINELKETKIPMKGARGDHDSNSYAELFGQPSMVFAFDTGLARIILLDSEKSASSNAEFLEKELKATKQPWKIVVTTTPLYTSPSEHEQDEDQTTALQPLLDKYGVDLVMWGDNHNYERIKFPNKHTVFIQSGTAGRSHYEFEGQINESIYQNDNDYGFTKILISPNSLMGQFISHSGKILDNFSIIK

> Delta3-3CBM32-2

MINLLKFASFSTWKSNQLKLGIFLITFLVLIFTSSVVQSFHTYAQSDPFGSSNTCSKLPVNGITASGADPFHPPSRAIDQNVNTRWSNLGLGSWIQIDLGQENVVCGVGINWHRGNERVNSFIISISKDGKTFTNVYSGKSDGTSLTEQNYNLQSKAGRFIRVMVSGNTQSNWISISEFKIYGYKALSESCVKSPISQVSAASSQIGFPSSNVVDNNLNSIWSNYGVGSSIQLDLGTSKSICSLEIAWYKGNERQNNFVVSISQDGKSYKTVLSTVSSGKSLSYEKYVFSDNLARYIKITINGNSQNNYASIAEIRAQVPSSGQSQVQCVDSHIQDAKTSGSQTGFPSTNVLDDNLDTRWSNNGVGSWIQLDLGTSNKICDINIAWYKGNERQNNFVISTSNDGIKFSNVFSSKSSGSTLNLEKYDIADTNARYIRIVVNGNTQNTYASITEISINIVSISGSSNYYIGAVGDWGSARNDNWEETVDLMINNKINLALGLGDYSYGSVSEFEPVVDTLKAAGIPFKGVQGNHDSSSYARLFGQPSMIHAFDAGQSRIIMLNTEESYSANTQFLENQLKNTKQPWKIVAMHNPLYSSPSNHPEEKELAGRLQPLFDQYGVDLVIYGHNHNYERIKLPDKPTVFIQAGTGGESHYDIKGSRSDGGVEFQDDNDYGFVKLTINSNTLSGQFISHGGKILDSFSMVK

**Appendix 1**

|  | **KO** | **Gene** | **Function** |
| --- | --- | --- | --- |
| **Central metabolism** | K10944 | amoA | pmoA-amoA; methane/ammonia monooxygenase subunit A [EC:1.14.18.3 1.14.99.39] |
|  | K10945 | amoB | pmoB-amoB; methane/ammonia monooxygenase subunit B |
|  | K10946 | amoC | pmoC-amoC; methane/ammonia monooxygenase subunit C |
|  | K01428 | ureC | ureC; urease subunit alpha [EC:3.5.1.5] |
|  | K01429 | ureB | ureB; urease subunit beta [EC:3.5.1.5] |
|  | K01430 | ureA | ureA; urease subunit gamma [EC:3.5.1.5] |
|  | K03187 | ureE | ureE; urease accessory protein |
|  | K03188 | ureF | ureF; urease accessory protein |
|  | K03189 | ureG | ureG; urease accessory protein |
|  | K03190 | ureD | ureD, ureH; urease accessory protein |
|  | K03320 | amt | amt, AMT, MEP; ammonium transporter, Amt family |
|  | K08717 | UT | utp; urea transporter |
|  | K03307 | SSS | TC.SSS; solute:Na+ symporter, SSS family |
|  | K00368 | nirK | nirK; nitrite reductase (NO-forming) [EC:1.7.2.1] |
| **MoCo** | K03637 | moaC | moaC, CNX3; cyclic pyranopterin monophosphate synthase [EC:4.6.1.17] |
|  | K03639 | moaA | moaA, CNX2; GTP 3',8-cyclase [EC:4.1.99.22] |
|  | K03750 | moeA | moeA; molybdopterin molybdotransferase [EC:2.10.1.1] |
|  | K03635 | moaE | MOCS2B, moaE; molybdopterin synthase catalytic subunit [EC:2.8.1.12] |
|  | K03636 | moaD | moaD, cysO; sulfur-carrier protein |
|  |  | MoOR | Molybdopterin oxidoreductase |
|  |  | MOSC | molybdenum cofactor sulfurase |
| **Msm** | K10112 | msmX | msmX, msmK, malK, sugC, ggtA, msiK; multiple sugar transport system ATP-binding protein [EC:7.5.2.-] |
|  | K02025 | MS.P | ABC.MS.P; multiple sugar transport system permease protein |
|  | K02026 | MS.P1 | ABC.MS.P1; multiple sugar transport system permease protein |
|  | K02027 | MS.S | ABC.MS.S; multiple sugar transport system substrate-binding protein |
| **Antiporters** | K07301 | yrbG | yrbG; cation:H+ antiporter |
|  | K03316 | CPA1 | TC.CPA1; monovalent cation:H+ antiporter, CPA1 family |
|  | K03455 | CPA2 | TC.KEF; monovalent cation:H+ antiporter-2, CPA2 family |
|  | K07300 | chaA | chaA, CAX; Ca2+:H+ antiporter |
|  | K06197 | chaB | chaB; cation transport regulator |
|  |  | nahD | ArsB/NhaD family transporter |
| **Transporters** | K03281 | CLC | clcA, clcB, CLC-E, CLC-F; chloride channel protein, CIC family |
|  | K03284 | corA | corA; magnesium transporter |
|  | K07507 | mgtC | mgtC; putative Mg2+ transporter-C (MgtC) family protein |
|  | K03498 | Trk | trkH, trkG, ktrB, ktrD; trk/ktr system potassium uptake protein |
|  | K10716 | kch | kch, trkA, mthK, pch; voltage-gated potassium channel |
|  | K01546 | kdpA | kdpA; potassium-transporting ATPase potassium-binding subunit |
|  | K01547 | kdpB | kdpB; potassium-transporting ATPase ATP-binding subunit [EC:7.2.2.6] |
|  | K01548 | kdpC | kdpC; potassium-transporting ATPase KdpC subunit |
| **Stress response** | K03686 | dnaJ | dnaJ; molecular chaperone DnaJ |
|  | K03687 | GrpE | GRPE; molecular chaperone GrpE |
|  | K13993 | HSP20 | HSP20; HSP20 family protein |
|  | K04564 | SOD2 | SOD2; superoxide dismutase, Fe-Mn family [EC:1.15.1.1] |
|  | K04043 | dnaK | dnaK, HSPA9; molecular chaperone DnaK |
|  | K05947 | MPGS | E2.4.1.217; mannosyl-3-phosphoglycerate synthase [EC:2.4.1.217] |
|  | K03671 | trxA | thioredoxin |
|  | K22447 | cct | cct, ths; archaeal chaperonin |
| **Glycolysis/**  **Gluconeogenesis** | K00150 | gapB | gap2, gapB; glyceraldehyde-3-phosphate dehydrogenase (NAD(P)+) (phosphorylating) [EC:1.2.1.59] |
|  | K00174 | korA | korA, oorA, oforA; 2-oxoglutarate/2-oxoacid ferredoxin oxidoreductase subunit alpha [EC:1.2.7.3 1.2.7.11] |
|  | K00175 | korB | korB, oorB, oforB; 2-oxoglutarate/2-oxoacid ferredoxin oxidoreductase subunit beta [EC:1.2.7.3 1.2.7.11] |
|  | K00927 | pgk | PGK, pgk; phosphoglycerate kinase [EC:2.7.2.3] |
|  | K01610 | pckA | pckA; phosphoenolpyruvate carboxykinase (ATP) [EC:4.1.1.49] |
|  | K01622 | FBA | fructose 1,6-bisphosphate aldolase/phosphatase [EC:4.1.2.13 3.1.3.11] |
|  | K01689 | eno | ENO, eno; enolase [EC:4.2.1.11] |
|  | K01803 | TPI | TPI, tpiA; triosephosphate isomerase (TIM) [EC:5.3.1.1] |
|  | K01895 | acs | ACSS1_2, acs; acetyl-CoA synthetase [EC:6.2.1.1] |
|  | K15634 | gpmB | gpmB; 2,3-bisphosphoglycerate-dependent phosphoglycerate mutase [EC:5.4.2.11] |
|  | K15635 | apgM | apgM; 2,3-bisphosphoglycerate-independent phosphoglycerate mutase [EC:5.4.2.12] |
|  | K15778 | pmm-pgm | pmm-pgm; phosphomannomutase / phosphoglucomutase [EC:5.4.2.8 5.4.2.2] |
|  | K01006 | pdK | pdK; pyruvate, orthophosphate dikinase [EC:2.7.9.1] |
| **3-hydroxypropionate/4-hydroxybutyrate (3HP/4HB)** | K18603 | K18603 | acetyl-CoA/propionyl-CoA carboxylase [EC:6.4.1.2 6.4.1.3] |
|  | K18604 | K18604 | acetyl-CoA/propionyl-CoA carboxylase [EC:6.4.1.2 6.4.1.3 2.1.3.15] |
|  | K18605 | K18605 | biotin carboxyl carrier protein |
|  | K18602 | K18602 | malonic semialdehyde reductase [EC:1.1.1.-] |
|  | K18594 | K18594 | 3-hydroxypropionyl-CoA synthetase (ADP-forming) [EC:6.2.1.-] |
|  | K15019 | K15019 | 3-hydroxypropionyl-coenzyme A dehydratase [EC:4.2.1.116] |
|  | K01848 | mcmA1 | methylmalonyl-CoA mutase, N-terminal domain [EC:5.4.99.2] |
|  | K01849 | mcmA2 | methylmalonyl-CoA mutase, C-terminal domain [EC:5.4.99.2] |
|  | K18593 | K18593 | 4-hydroxybutyrate---CoA ligase (ADP-forming) [EC:6.2.1.56] |
|  | K14534 | abfD | 4-hydroxybutyryl-CoA dehydratase / vinylacetyl-CoA-Delta-isomerase [EC:4.2.1.120 5.3.3.3] |
|  | K15016 | K15016 | enoyl-CoA hydratase / 3-hydroxyacyl-CoA dehydrogenase [EC:4.2.1.17 1.1.1.35] |
|  | K00626 | atoB | ACAT, atoB; acetyl-CoA C-acetyltransferase [EC:2.3.1.9] |
| **Krebs cycle** | K01647 | CS | CS, gltA; citrate synthase [EC:2.3.3.1] |
|  | K01681 | ACO | ACO, acnA; aconitate hydratase [EC:4.2.1.3] |
|  | K00030 | IDH3 | isocitrate dehydrogenase (NAD+) [EC:1.1.1.41] |
|  | K00174 | korA | korA, oorA, oforA; 2-oxoglutarate/2-oxoacid ferredoxin oxidoreductase subunit alpha [EC:1.2.7.3 1.2.7.11] |
|  | K00175 | korB | korB, oorB, oforB; 2-oxoglutarate/2-oxoacid ferredoxin oxidoreductase subunit beta [EC:1.2.7.3 1.2.7.11] |
|  | K01902 | sucD | succinyl-CoA synthetase alpha subunit [EC:6.2.1.5] |
|  | K01903 | sucC | succinyl-CoA synthetase beta subunit [EC:6.2.1.5] |
|  | K00239 | sdhA | sdhA, frdA; succinate dehydrogenase flavoprotein subunit [EC:1.3.5.1] |
|  | K00240 | sdhB | sdhB, frdB; succinate dehydrogenase iron-sulfur subunit [EC:1.3.5.1] |
|  | K00241 | sdhC | sdhC, frdC; succinate dehydrogenase cytochrome b subunit |
|  | K00242 | sdhD | sdhD, frdD; succinate dehydrogenase membrane anchor subunit |
|  | K00024 | mdh | malate dehydrogenase [EC:1.1.1.37] |
|  |  |  |  |
|  |  |  |  |

(https://www.kegg.jp/)

**Appendix 2**

| **GH family** | **Activities in Family** |
| --- | --- |
| **CBM32** | Binding to galactose and lactose has been demonstrated for the module of Micromonospora viridifaciens sialidase (PMID: 16239725). Binding to polygalacturonic acid has been shown for a Yersinia member (PMID: 17292916). Binding to LacNAc (β-D-galactosyl-1,4-β-D-N-acetylglucosamine) has been shown for an N-acetylglucosaminidase from Clostridium perfingens (PMID: 16990278). |
| **CE4** | acetyl xylan esterase (EC 3.1.1.72); chitin deacetylase (EC 3.5.1.41); chitooligosaccharide deacetylase (EC 3.5.1.-); peptidoglycan GlcNAc deacetylase (EC 3.5.1.-); peptidoglycan N-acetylmuramic acid deacetylase (EC 3.5.1.-). |
| **CE14** | N-acetyl-1-D-myo-inosityl-2-amino-2-deoxy-α-D-glucopyranoside deacetylase (EC 3.5.1.89); diacetylchitobiose deacetylase (EC 3.5.1.-); mycothiol S-conjugate amidase (EC 3.5.1.-); chitin disaccharide deacetylase (NRE) (EC 3.5.1.136) |
| **GH5** | beta-glucosidase (EC 3.2.1.21); beta-galactosidase (EC 3.2.1.23); beta-mannosidase (EC 3.2.1.25); beta-glucuronidase (EC 3.2.1.31); beta-xylosidase (EC 3.2.1.37); beta-D-fucosidase (EC 3.2.1.38); phlorizin hydrolase (EC 3.2.1.62); exo-beta-1,4-glucanase (EC 3.2.1.74); 6-phospho-beta-galactosidase (EC 3.2.1.85); 6-phospho-beta-glucosidase (EC 3.2.1.86); strictosidine beta-glucosidase (EC 3.2.1.105); lactase (EC 3.2.1.108); amygdalin beta-glucosidase (EC 3.2.1.117); prunasin beta-glucosidase (EC 3.2.1.118); vicianin hydrolase (EC 3.2.1.119); raucaffricine beta-glucosidase (EC 3.2.1.125); thioglucosidase (EC 3.2.1.147); beta-primeverosidase (EC 3.2.1.149); isoflavonoid 7-O-beta-apiosyl-beta-glucosidase (EC 3.2.1.161); ABA-specific beta-glucosidase (EC 3.2.1.175); DIMBOA beta-glucosidase (EC 3.2.1.182); beta-glycosidase (EC 3.2.1.-); hydroxyisourate hydrolase (EC 3.-.-.-); beta-rutinosidase /alpha-L-rhamnose-(1,6)-beta-D-glucosidase (EC 3.2.1.-) |
| **GH38** | α-mannosidase (EC 3.2.1.24); mannosyl-oligosaccharide α-1,2-mannosidase (EC 3.2.1.113); mannosyl-oligosaccharide α-1,3-1,6-mannosidase (EC 3.2.1.114); mannosyl-oligosaccharide α-1,3-mannosidase (EC 3.2.1.207); mannosyl-oligosaccharide α-1,6-mannosidase / exo-α-1,6-mannosidase (EC 3.2.1.163) |
| **GH130** | β-1,4-mannosylglucose phosphorylase (EC 2.4.1.281); β-1,4-mannooligosaccharide phosphorylase (EC 2.4.1.319); β-1,4-mannosyl-N-acetyl-glucosamine phosphorylase (EC 2.4.1.320); β-1,2-mannobiose phosphorylase (EC 2.4.1.339); β-1,2-oligomannan phosphorylase (EC 2.4.1.340); β-1,2-mannosidase (EC 3.2.1.-); β-1,3-oligomannan phosphorylase (EC 2.4.1.-) |
| **GH133** | amylo-α-1,6-glucosidase (EC 3.2.1.33) |

(http://www.cazy.org/)

**Appendix 3**


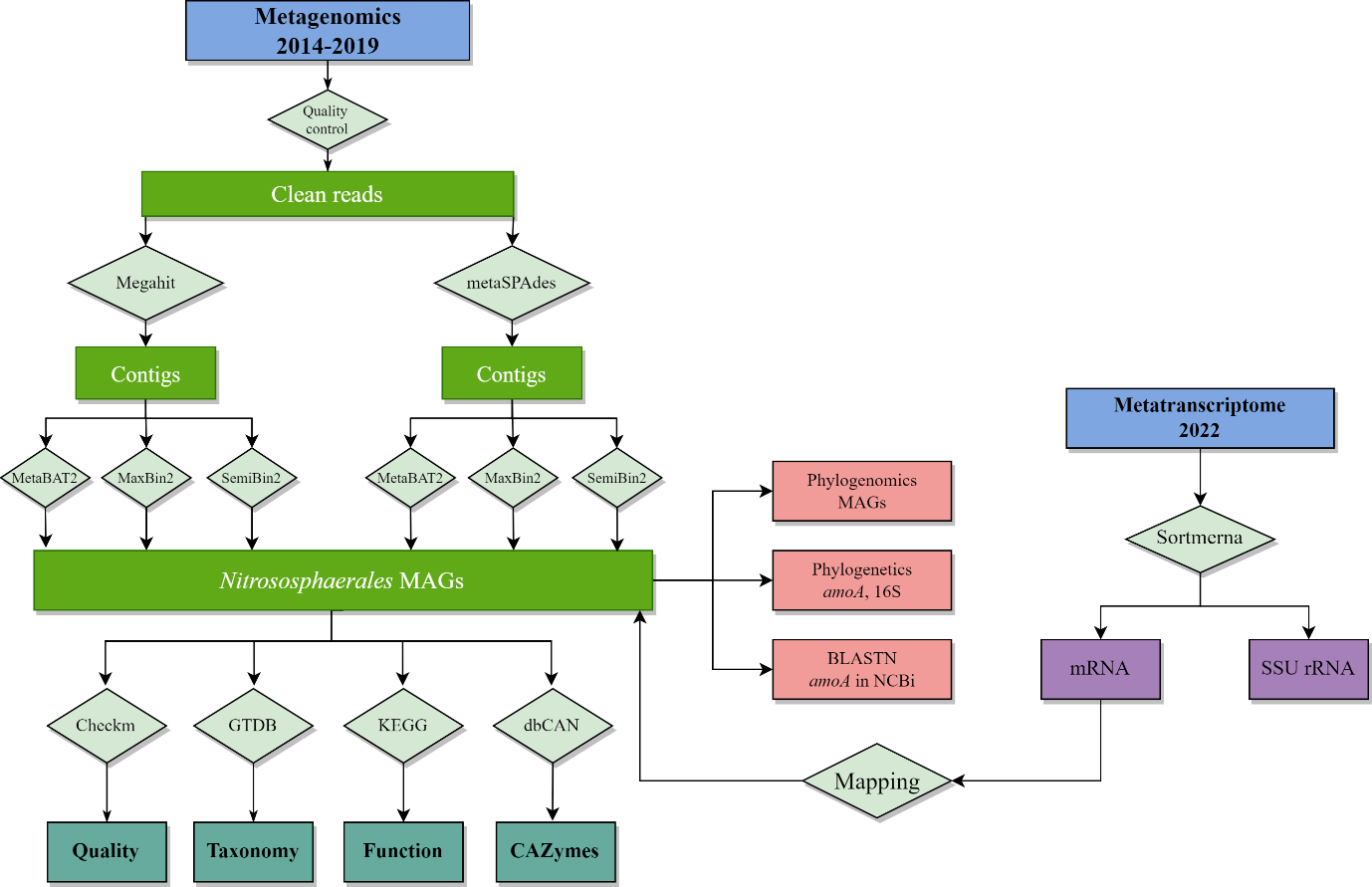

Supplement: ISME_Supplemental_Figures_R3_wrae086 [file isme_supplemental_figures_r3_wrae086.docx]
